# BGOA-TVG: Binary Grasshopper Optimization Algorithm with Time-Varying Gaussian Transfer Functions for Feature Selection

**DOI:** 10.3390/biomimetics9030187

**Published:** 2024-03-20

**Authors:** Mengjun Li, Qifang Luo, Yongquan Zhou

**Affiliations:** 1 College of Artificial Intelligence, Guangxi Minzu University, Nanning 530006, China; lelejingxuan@163.com (M.L.); l.qf@163.com (Q.L.); 2 Guangxi Key Laboratories of Hybrid Computation and IC Design Analysis, Nanning 530006, China; 3 Faculty of Information Science and Technology, Universiti Kebangsaan Malaysia, Bangi 43600, Selangor, Malaysia

**Keywords:** binary grasshopper optimization algorithm, time-varying Gaussian transfer function, UCI dataset, DEAP dataset, EPILEPSY dataset, feature selection, metaheuristic

## Abstract

Feature selection aims to select crucial features to improve classification accuracy in machine learning and data mining. In this paper, a new binary grasshopper optimization algorithm using time-varying Gaussian transfer functions (BGOA-TVG) is proposed for feature selection. Compared with the traditional S-shaped and V-shaped transfer functions, the proposed Gaussian time-varying transfer functions have the characteristics of a fast convergence speed and a strong global search capability to convert a continuous search space to a binary one. The BGOA-TVG is tested and compared to S-shaped and V-shaped binary grasshopper optimization algorithms and five state-of-the-art swarm intelligence algorithms for feature selection. The experimental results show that the BGOA-TVG has better performance in UCI, DEAP, and EPILEPSY datasets for feature selection.

## 1. Introduction

Researchers from all over the world have been paying more and more attention to how to handle massive datasets in recent years, thanks to data exploration. These databases can contain duplicate and pointless features. Thus, in order to solve this issue and increase the effectiveness of both supervised and unsupervised learning algorithms, feature selection is crucial [1,2,3]. It is obviously a challenging task to find the best subset of features in high-dimensional feature datasets because there are $2^{N-1}$ feature subsets in a dataset with *N* features. Traditional mathematical techniques cannot produce the desired outcome in an acceptable amount of time; hence, meta-heuristic algorithms are frequently used for feature selection issues.

Metaheuristic algorithms have been successfully used to solve complex engineering and science computation problems, such as function optimization [4,5,6], engineering optimization [7,8,9], and feature selection problems [10]. Researchers have proposed binary metaheuristic algorithms or improved versions for feature selection, such as binary swarm optimization (BPSO) [11], the binary artificial bee colony (BABC) [12], the binary gravitational search algorithm (BGSA) [13], binary grey wolf optimization (BGWO) [14], the binary salp swarm algorithm (BSSA) [15], the binary bat algorithm (BBA) [16], the binary whale optimization algorithm (BWOA) [17], binary spotted hyena optimization (BSHO) [18], binary emperor penguin optimization (BEPO) [19], binary Harris hawks optimization (BHHO) [20], binary equilibrium optimization (BEO) [21], binary atom search optimization (BASO) [22], the binary dragonfly algorithm (BDA) [23], the binary jaya algorithm (BJA) [24], binary coronavirus herd immunity optimization (BCHIO) [25], the binary butterfly optimization algorithm (BBOA) [26], binary black widow optimization (BBWO) [27], the binary slime mould algorithm (BSMA) [28], binary golden eagle optimization (BGEO) [29], and so on.

To find the best solution to complex optimization problems, metaheuristics are employed. A system of solutions that develops over many iterations utilizing a set of rules or mathematical equations can be used by several agents to facilitate the search process. These iterations continue until the result satisfies a set of predetermined requirements. This final solution (the near-optimal solution) is called the optimal solution, and the system is considered to have reached a state of convergence [30]. 

In contrast to exact methods that find optimal solutions but require a long computational time, heuristic methods find near-optimal solutions quite quickly [31]. However, most of these methods are problem-specific. As the word “meta” in metaheuristic methods indicates, metaheuristics are one level higher than heuristics. Metaheuristics have been very successful because they have the potential to provide solutions at an acceptable computational cost. By mixing good heuristics with classical metaheuristics, very good solutions can be obtained for many real-world problems.

Binary particle swarm optimization (BPSO) and its variants have been widely used in the FS problem. In 2020, the authors of ref. [32] proposed self-adaptive PSO with a local search strategy to find less-correlated feature subsets. The authors of ref. [33] proposed an improved version of the SSA algorithm called the ISSA to solve the FS problem. Furthermore, a binary chaotic horse herd optimization algorithm for feature selection (BCHOAFS) was proposed by Esin Ayşe Zaimoğlu [34]. The authors of ref. [35] used four families of transfer functions in the binary AOA (BAOA) to test 10 low-dimensional and 10 high-dimensional datasets. In feature selection problems, several wrapping-based algorithms using binary meta-heuristic algorithms have been proposed. The S-shaped and V-shaped transfer functions are mostly used, such as modified binary SSA (MBSSA) [36].

In recent years, to increase the efficiency of transfer functions, the time-varying S-shaped and V-shaped transfer functions have been proposed and applied to the binary dragonfly algorithm (BDA) [37] and BMPA [38]. The results showed that the time-varying S-shaped transfer function has a better performance than the V-shaped one. Besides the single-objective algorithm, the muti-objective algorithm also plays an important role in the feature selection problem. Multi-objective whale algorithm optimization (WOA) [39] was proposed for data classification for which filter and wrapper fitness functions were simultaneously optimized. Another paper studies a multi-label feature selection algorithm using improved multi-objective particle swarm optimization (PSO) [40], with the purpose of searching for a Pareto set of non-dominated solutions (feature subsets). Liu proposes a novel feature selection method utilizing a filtered and supported sequential forward search technique called multi-objective ant colony optimization (MOACO) in the context of support vector machines (SVMs) [41] to solve the feature selection problem. A new method known as improved multi-objective salp swarm algorithm (IMOSSA) is tested for a feature selection task [42]. Also, other kinds of transfer functions are used in feature selection problems. An automata-based improved BEO [43] (AIEOU) used a U-shaped transfer function to select the best subset of features. This algorithm applied both learning-based automata and adaptive β-hill climbing (AβHC) to find the best parameters to form a better equilibrium pool. Many binary metaheuristic algorithms have been introduced to select the best subset of features. However, due to the importance of feature selection in many fields, it is necessary to design algorithms that can obtain a higher accuracy with a smaller subset of features.

An important step in the feature selection problem is mapping a continuous space to binary ones, and the transfer function plays a significant role in this process. Moreover, using transfer functions is one of the easiest ways to convert an algorithm from continuous to binary without modifying its structure. The shapes of transfer functions are classified into two families: S-shaped and V-shaped [44]. The S-shaped transfer function is a transformation function that increases monotonically within the [0, 1] interval, and its main attribute is that it is asymmetric and has a single fixed value throughout its entire area. The V-shaped transformation function is symmetric and has two numbers with equal values in the [0, 1] interval, which enhances the diversity of the population in the region within the range.

A common drawback of common transfer functions used in binary algorithms is that they do not explore and develop evolutionarily during the search for the optimal solution; that is, the process to obtain a solution involves changing the probability of the parameter values in a nonadaptive manner. Thus, they have poor exploration or exploitation and are static functions which cannot change as time goes on.

In this paper, a powerful transfer function is proposed to convert continuous search spaces to binary ones. The transfer function is dynamic during this process, which can enhance the search ability of the BGOA in the exploration phase. Then, the transfer function gradually changes while the proposed algorithm switches from exploration to exploitation and finally reaches good result in the end, the *K*-nearest neighbors (KNN) algorithm is applied to classify it.

The main contributions for this paper can be summarized as follows:

1. A time-varying Gaussian transfer function is introduced.
2. A new binary grasshopper optimization algorithm based on time-varying Gaussian transfer functions (BGOA-TVG) is proposed.
3. The BGOA-TVG achieves a balance between its exploration and exploitation capabilities and improves the convergence speed of the algorithm.
4. The BGOA-TVG can effectively deal with high-dimensional feature selection problems.
5. Compared with proposed binary metaheuristic optimization algorithms in recent years, the excellent performance of the BGOA-TVG is verified.

The rest of the paper is organized as follows: Section 2 presents a brief introduction to the feature selection problem. In Section 3, the basic grasshopper optimization algorithm is discussed. The enhanced transfer function is presented in Section 4. Section 5 shows the results of the test. In Section 6, the proposed method is demonstrated within the EEG analysis field. Finally, Section 7 concludes the paper and suggests some directions for future studies.

## 2. Feature Selection Problem

The feature selection problem is an NP-hard optimization problem [45], in which as the features of a dataset increase, the search space of the problem exponentially grows. It is a useful way to find a relevant subset of fewer features from an initial dataset to reduce dimensions and training times [46]. However, traditional mathematic methods cannot solve high-dimensional feature selection problems in a reasonable time, and according to tests, metaheuristic algorithms are better at finding subsets of features [47,48,49,50,51,52,53,54]. There are three selection strategies in feature selection: wrapper-based, filter-based, and hybrid filter–wrapper-based methods [55]. The precision of the learning algorithm in the wrapper-based strategy creates the optimum subset. The chosen subset in the filter-based method is unrelated to the learning process. These methods are combined in the hybrid approach. The wrapper-based method outperforms the others in terms of accuracy, but it requires more CPU resources and a longer testing time. The authors of [56,57,58] provided a new method to extract optimal feature subset to enhance accuracy of the calculation. Others have proposed correlation feature selection [59] and in-depth analyses on the usage of searching methods like the best-first, greedy step-wise, genetic, linear forward selection, and rank searches [60].

A feature selection module is applied prior to the classification method to optimize efficiency and precision by eliminating irrelevant features and to reduce the time complexity to find the classification to which a document belongs [61].

The most important thing in feature extraction is to extract subsets and determine whether to select an element in the set according to the accuracy rate. In the binary algorithm, individuals traverse a set and display whether to select an element using 0 s and 1 s. In Equation (1), N is the number of the dataset and SF is a subset selected by the algorithms; in the binary algorithm, it is selected by the value of an individual.

Feature selection is a multi-objective optimization problem for which we aim to minimize the subset of the selected features and maximize the classification accuracy, which is described as a fitness function as follows:

(1) $$Fitness=\alpha \times Err+\beta \times \frac{SF}{N}$$

where Err is the resulting classification error. SF is the number of selected features of the subset, and N is the total number of features of the dataset. $\frac{SF}{N}$ is the feature selection ratio of the subset to the total set. α and β are parameters in the interval of [0, 1] and α=1−β.

## 3. Grasshopper Optimization Algorithm (GOA)

The grasshopper optimization algorithm is a population-based swarm intelligence algorithm introduced by Mirjalili et al. in 2017 [62], which models the behaviour of grasshopper swarms in nature. There are two essential phases in this algorithm: the exploration and exploitation of the search space. Through social interactions during the food search process, the swarm of grasshoppers changes between the phases. The swarm moves slowly and goes a small distance in the larval stage. In contrast, the swarm moves quickly and goes a large distance in adulthood.

There are three evolutionary operators in the position-updating process of individuals in swarms [62]: the social interaction operator, $S_{i}$ in Equation (2); the gravity force operator, $G_{i}$ in Equation (2); and the wind advection operator, $A_{i}$ in Equation (2). The movement of individuals in the swarm is describes as follows:

(2) $$X_{i}=S_{i}+G_{i}+A_{i}$$

where $X_{i}$ defines the position of the $i^{th}$ grasshopper.

(3) $$S_{i}=\sum_{\begin{array}{c} j=1\\ j\neq i \end{array}}^{N}S\left(\left|X_{j}-X_{i}\right|\right)\frac{X_{j}-X_{i}}{d_{ij}}$$

where *N* is the number of grasshoppers in the swarm, $d_{ij}$ represents the distance between the $i^{th}$ and the $j^{th}$ grasshopper, S is a function that defines the strength of the social forces and is calculated as shown in Equation (4), and $\frac{X_{j}-X_{i}}{d_{ij}}$ is the unit vector from the $i^{th}$ grasshopper to the $j^{th}$.

(4) $$S\left(r\right)=fe^{\frac{-r}{l}}-e^{-r}$$

where f and l are two constants that indicate the intensity of attraction and the attraction length scale, respectively, and r is a real value.

$G_{i}$ in Equation (2) is calculated as shown in Equation (5) below:

(5) $$G_{i}=-g\times \vec{e_{g}}$$

where g is the gravitational constant, and $\vec{e_{g}}$ shows a unity vector towards the center of the earth. The effect of an individual’s flight to overcome gravity is represented by the symbol preceding it.

$A_{i}$ in Equation (2) is calculated as shown in Equation (6) below:

(6) $$A_{i}=u\times \vec{e_{w}}$$

where u is a constant drift, and $\vec{e_{w}}$ is a unity vector in the direction of the wind.

Equation (2) can be expanded to Equation (7) as follows:

(7) $$X_{i}=\sum_{\begin{array}{c} j=1\\ j\neq i \end{array}}^{N}S\left(\left|X_{j}-X_{i}\right|\right)\frac{X_{j}-X_{i}}{d_{ij}}-g\times \vec{e_{g}}+u\times \vec{e_{w}}$$

However, the mathematical model using Equation (7) cannot be used directly to solve optimization problems, mainly because the grasshoppers quickly reach their comfort zone and the swarm does not converge to a specified point, according to a test in ref. [62]. The author of the GOA algorithm suggested a modified version of Equation (7) as shown in Equation (8) to solve optimization problems [62], where the gravity operator is unconsidered, the gravity factor is set to 0, and the wind direction is always defined as moving towards a target. Accordingly, Equation (2) becomes Equation (8) as follows:

(8) $$X_{i}^{d}=c\left(\sum_{\begin{array}{c} j=1\\ j\neq i \end{array}}^{N}c\frac{{ub}_{d}-{lb}_{d}}{2}S(|X_{j}-X_{i}|)\frac{X_{j}-X_{i}}{d_{ij}}\right)+T_{d}$$

where ${ub}_{d}$ is the upper bound in the dth dimension, and ${lb}_{d}$ is the lower bound in the $d^{th}$ dimension. $T_{d}$ is the value of the $d^{th}$ dimension in the target (the best solution found so far). The coefficient c reduces the comfort zone proportional to the number of iterations and is calculated in Equation (9) as follows.

(9) $$c=C_{max}-l\frac{C_{max}-C_{min}}{L}$$

where $C_{max}$ is the maximum value, $C_{min}$ is the minimum value, l indicates the current iteration, and L is the maximum number of iterations. In ref. [62], they use $C_{max}=1$ and $C_{min}=0.00001$. Equation (8) shows that the next position of a grasshopper is defined based on its current position, the position of all other grasshoppers, and the position of the target. Algorithm 1 shows the pseudocode of the GOA algorithm.

## 4. Our Proposed BGOA-TVG Method

A binary search space is commonly considered as a hypercube [63]. The space is four-dimensional, which is formed by moving three-dimensional objects. In this search space, the search agents of the binary optimization algorithm can only move to nearer and farther corners of this hypercube by flipping various numbers of bits. Therefore, to design the binary version of the GOA, the concepts of the velocity- and position-updating process should be modified.

In the continuous version of the GOA, the swarm of grasshoppers moves around the search space by utilizing direction vectors, and the value of position is in the continuous real domain. In the binary space, due to dealing with only two numbers (“0” and “1”), the position cannot be updated using Equation (6). The way to change the position and velocity is outlined below.

In binary spaces, position updating means switching between “0” and “1” values. This switching should be based on the probability of updating the binary solution’s elements from 0 to 1 and vice versa. The main problem here is how to change the concept of velocity in the real world to a binary space. 

In order to achieve this, a transfer function is important to map velocity values to probability values to update the positions. In other words, a transfer function defines the probability of changing a position element from 0 to 1 and vice versa. In general, transfer functions force predators to move in a binary space. According to ref. [64], the following concepts should be taken into consideration when selecting a transfer function in order to map velocity values to probability values:

(1) The range of a transfer function should be bounded in the interval [0, 1], as this represents the probability that a particle will change its position.

(2) A transfer function should have a high probability of changing position for large absolute values of velocity. Particles with large absolute values for their velocities are probably far from the best solution, so they should switch their positions in the next iteration.

(3) A transfer function should also have a small probability of changing position for small absolute values of velocity.

(4) The return value of a transfer function should increase as the velocity rises. Particles that are moving away from the best solution should have a higher probability of changing their position vectors in order to return to their previous positions.

(5) The return value of a transfer function should decrease as the velocity is reduced.

(6) These concepts guarantee that a transfer function is able to map the process of searching from a continuous search space to a binary search space while preserving simi-lar concepts of the search for a particular evolutionary algorithm. The GOA is simulated by PSO, the changed part in Equation (8). Defined as ΔX, Equation (10) is analogous to the velocity vector (step) in PSO [65]. The transfer function defines the probability of updating the binary solution’s elements from 0 to 1 and vice versa. In the BGOA, the probability of changing the positions of elements is based on the step vector values.

(10) $$\Delta X({{ub}_{d}{,lb}_{d},X}_{i},X_{j})=c_{1}\left(\sum_{\begin{array}{c} j=1\\ j\neq i \end{array}}^{N}c_{2}\frac{{ub}_{d}-{lb}_{d}}{2}S(|X_{j}-X_{i}|)\frac{X_{j}-X_{i}}{d_{ij}}\right)$$

New time-varying transfer functions are proposed to enhance the ability of the BGOA in the search space. Algorithm 1 shows the pseudocode of the BGOA-TVG. The first transfer function (time-varying sin) is proposed to convert positions in the continuous space into the binary search space. The position is in the range of [${lb}_{d},{ub}_{d}$]. The binary position is mapped as follows:

(11) $$Time-varying\left(position,\alpha \right)=\left|\sin\left(\frac{position}{\alpha }\right)\right|$$

(12) $$Binaryposition=\left\{\begin{array}{c} 1\;\;\;\;if\;rand<Time-varying\left(position,\alpha \right)\\ 0\;\;\;\;if\;rand\geq Time-varying\left(position,\alpha \right) \end{array}\right.$$

where α is in the range of [$\alpha_{min}$, $\alpha_{max}$], and the linear increase in Equation (11) switches the algorithm smoothly from the exploration to the exploitation phases.

(13) $$\alpha =\alpha_{min}+iteration\left(\alpha_{max}-\alpha_{min}\right)/Max\_Iterations$$

**Figure 1:**
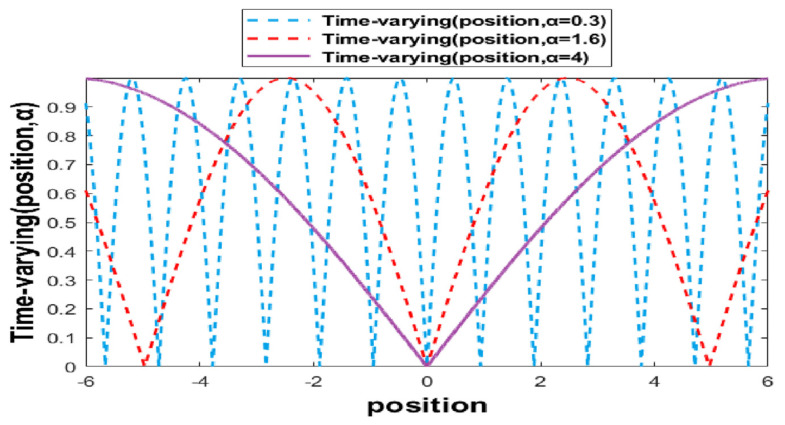
Time-varying transfer sine function.

Figure 1 shows the time-varying transfer function. It enhances the capability of the exploration in the first phase, as shown by the blue curve in Figure 1. In this phase, the diversity is extremely high, so the swarm can search all of the space. The red curve shows the phase between exploration and exploitation, which has a lower level of diversity than the first phase and searches more around the good solutions. The last phase, shown by the purple curve, changes slowly for the last iterations.

**Figure 2:**
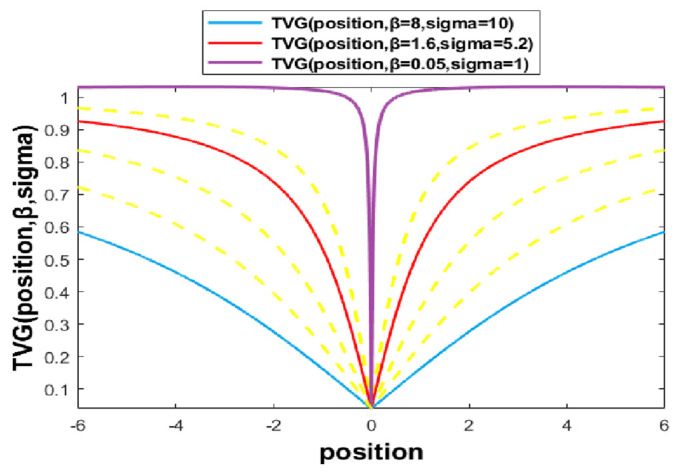
Time-varying V-shaped transfer function mixed with Gaussian function.

To avoid the local optima, the GOA uses Equation (8) to update the best solution. In the BGOA-TVG, a new time-varying V-shaped transfer function combined with a Gaussian mutation is proposed, as shown in Figure 2. The binary solutions are generated based on the TVG as shown in Equation (14) and are defined as follows:

(14) $$TVG\left(position,\beta ,sigma\right)=|\frac{2}{\pi }arctan(\frac{\pi }{2}position/\beta )|+Gaussian\left(0,sigma\right)$$

(15) $$\beta =\beta_{max}-\frac{iteration\left(\beta_{max}-\beta_{min}\right)}{{Max}_{Iterations}}$$

(16) $$sigma={sigma}_{max}-iteration\left({sigma}_{max}-{sigma}_{min}\right)/Max\_Iterations$$

where β is in the range of [0.05, 10], and sigma is in the range of [0.01, 10] to switch efficiently from the exploration to the exploitation phases over time.

(17) $$Binaryposition=\left\{\begin{array}{c} \neg Binaryposition\;\;\;\;if\;rand<TVG\left(position,\beta ,sigma\right)\\ Binaryposition\;\;\;\;\;\;\;\;if\;rand\geq TVG\left(position,\beta ,sigma\right) \end{array}\right.$$

In Figure 2, the blue curve is the initial status of the combined function, which has both exploration and exploitation, and the purple curve is the last status, which has maximal exploitation. Because the parameters β and sigma in the function are constantly changing with each iteration, the intermediate conversion function is also constantly changing. The yellow curves depict diverse scenarios characterized by varying parameters.

Algorithm 1 shows the pseudocode of the BGOA-TVG algorithm. In ref. [65], it showed that normalizing the distance of grasshoppers in [1, 4], individuals can have both attraction and repulsion forces, which balance exploration and exploitation in the algorithm. Hence, we set the distance of individuals within the closed interval [1, 4].

**Figure 3:**
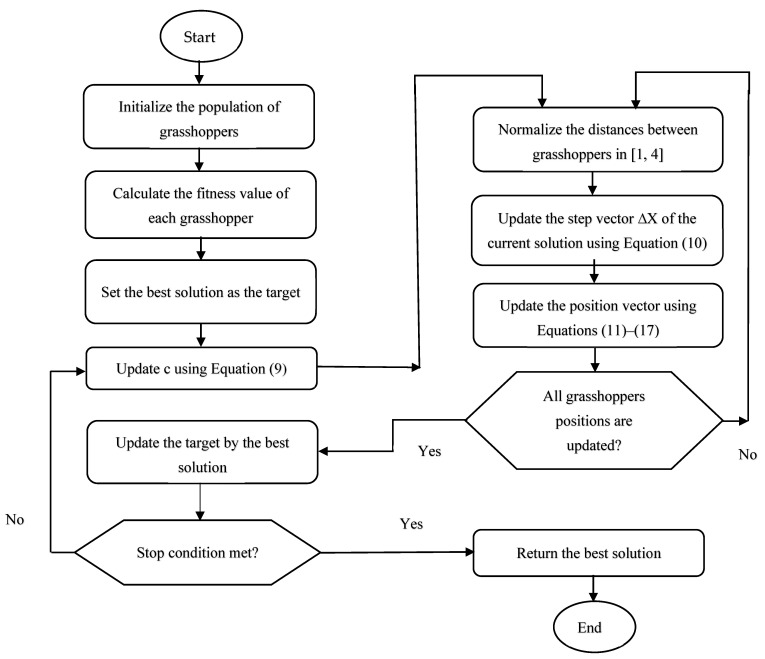
Flowchart of the proposed algorithm.

Figure 3 shows the flowchart of the proposed algorithm.
**Algorithm 1:** Pseudocode of the BGOA-TVG algorithm.Initialize $C_{max}$, $C_{min}$, and *Max_Iterations*Initialize a population of solutions $X_{i}$ (i = 1, 2, …, *n*)Evaluate each solution in the populationSet *T* as the best solution**While** (*t* < *Max_Iterations*)     Update c using Equation (9)     **For** each search agent          Normalize the distances between grasshoppers in [1, 4]          Update the step vector Δ*X* of the current solution using Equation (10)          **For**
*i* = 1: dim                Use Equation (8) to obtain the current position                Use Equations (10)–(13) to obtain the binary position                Use Equations (14)–(17) to obtain the final position                Calculate α,β,sigma based on Equations (13), (15), and (16)          End          Reevaluate the fitness of each individual in the population          If there is a better solution, replace T with it          Update T     **End****End****Return** *T*

### Computational Complexity

The proposed transfer functions do not change the computational complexity of the algorithm during each iteration. Moreover, the core program of the grasshopper optimization algorithm is to find the current optimal value in a loop. Factors that affect the overall complexity include the population number, number of individuals, and number of iterations. Therefore, the maximum of the BGOA-TVG is *O* (Max_Iteration×N×D), where *D* shows the number of dimensions.

## 5. Experimental Results and Discussion

### 5.1. Experimental Simulation Platform

For this experiment, we used Windows10 on a computer with the following specifications: a main frequency of 3.30 GHz; a memory of 16.0 GB; and an Inter(R) Core (TM)i3_6100 CPU. All algorithm codes were run in MATLAB2022a.

### 5.2. UCI Datasets

For this section, we selected 10 University of California at Irvine (UCI) datasets with different characteristics to verify the BGOA-TVG from different perspectives in terms of its performance. The name of the dataset, number of features, and number of instances the dataset has been used are shown in Table 1.

### 5.3. Parameter Settings

In order to verify the feasibility and effectiveness of the BGOA-TVG, we adopted some binary meta-heuristic algorithms: the BDA [23], BHHO [20], BPSO [11], BGWO [14], the BGBO [66], the BWOA [17], BGA [67], BBOA [68] and the BGOA-TVG. To make a fair comparison, the population size of the seven algorithms is set to 40, and the number of iterations is set to 100. Table 2 shows the main parameter settings of the seven algorithms.

### 5.4. Evaluation Criteria

The experimental results are evaluated in terms of the following criteria:

(1) Average fitness function

The datasets are tested dependently 30 times, and the average fitness shows the stability of the proposed algorithm, as calculated in Equation (18):

(18) $$Mean=\frac{1}{N}\sum_{i=1}^{N}{fitness}_{i}$$

where *N* is the number of runs of the optimization algorithm, and ${fitness}_{i}$ is the optimal solution resulting from the $i^{th}$ run.

(2) Average classification accuracy

The result is formulated in Equation (19) as follows:

(19) $$Acc=\frac{1}{N}\sum_{i=1}^{N}{Acc}_{i}$$

where *N* is the total number of runs of the proposed algorithm to select the subset. ${Acc}_{i}$ is the accuracy of the best solution from the $i^{th}$ run.

(3) Average feature selection size

This criterion can be calculated as in Equation (20), and the result is shown in Table 3.

(20) $$Afss=\frac{1}{N}\sum_{i=1}^{N}\frac{size(i)}{D}$$

where *N* is the total number of runs of the proposed algorithm to select the subset. size(i) returns the number of features selected in the best solution from the $i^{th}$ run, and D is the size of the original dataset.

The results of the average classification accuracy are shown in Table 3. Among them, the BGOA-TVG achieved the best results. It managed to reach the highest precision in nine datasets, and in addition, it had the highest precision value among all the methods in five datasets, so it came in first place in the overall ranking.

The performance of the proposed algorithm will be more clearly demonstrated by comparing it with the other 7 algorithms, as depicted in Figure 4, Figure 5, Figure 6, Figure 7, Figure 8, Figure 9, Figure 10, Figure 11, Figure 12 and Figure 13. 

### 5.5. Different Transfer Functions

**Figure 14:**
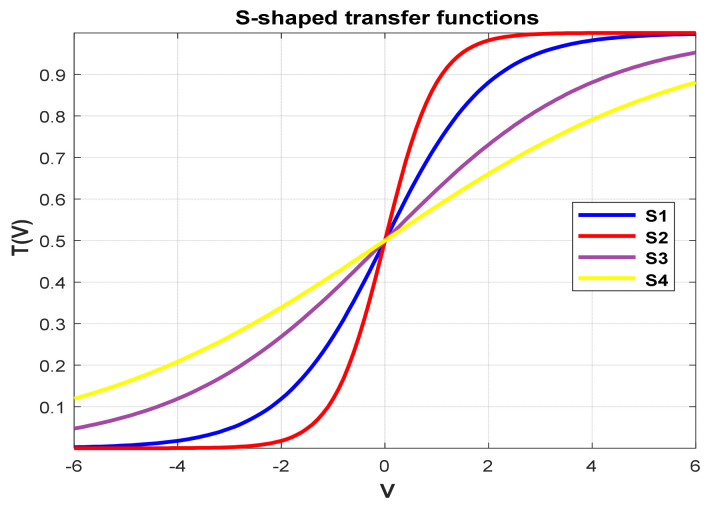
S-shaped.

**Figure 15:**
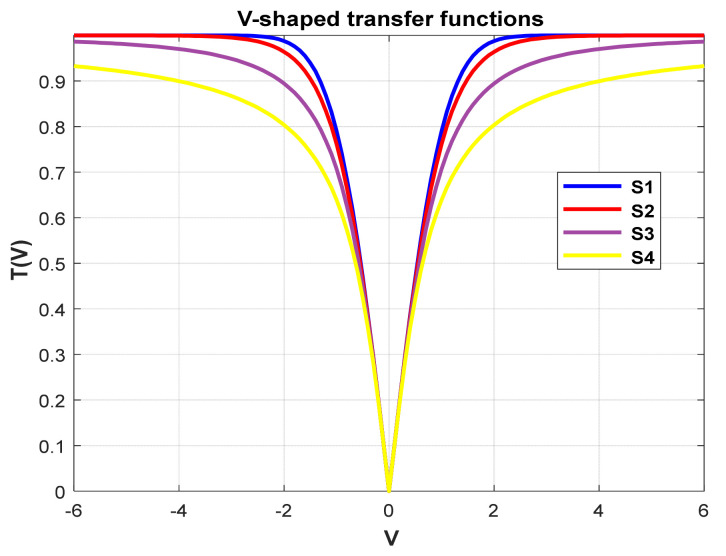
V-shaped.

**Figure 16:**
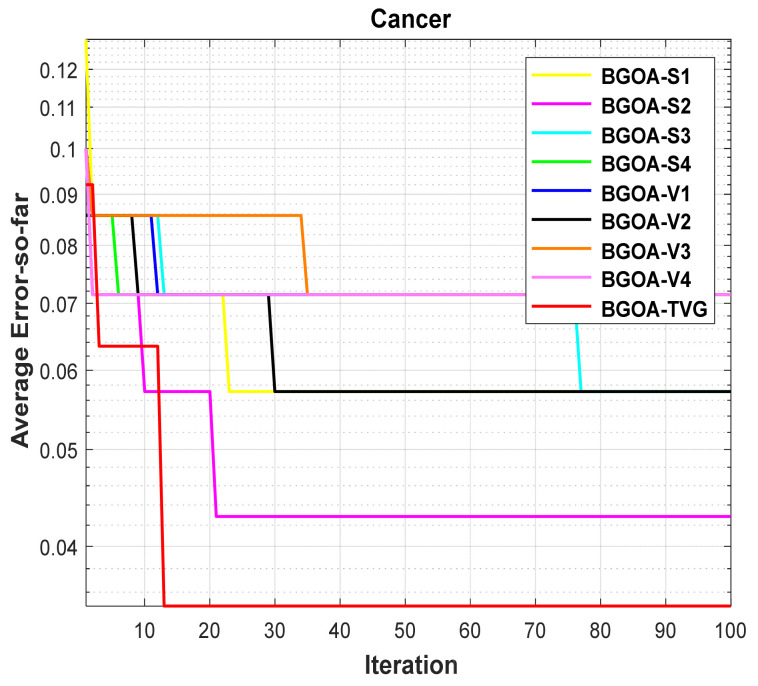
Cancer.

**Figure 17:**
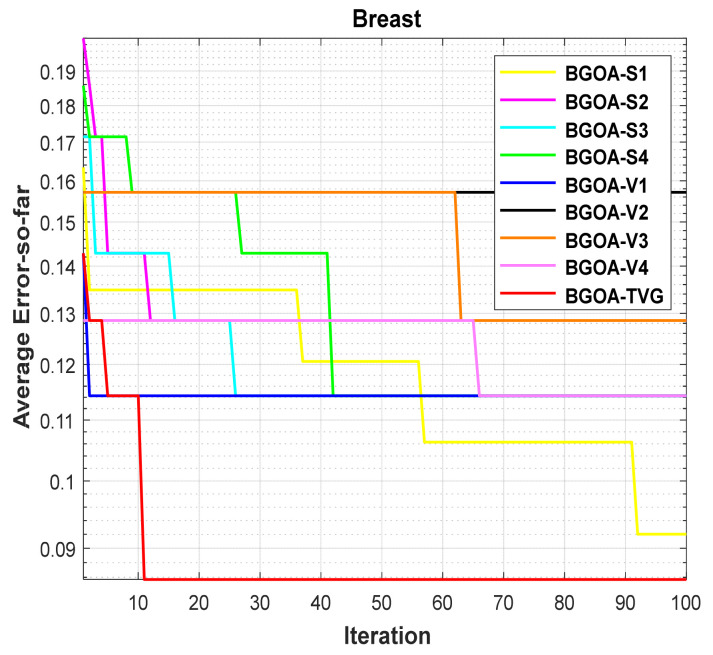
Breast.

**Figure 18:**
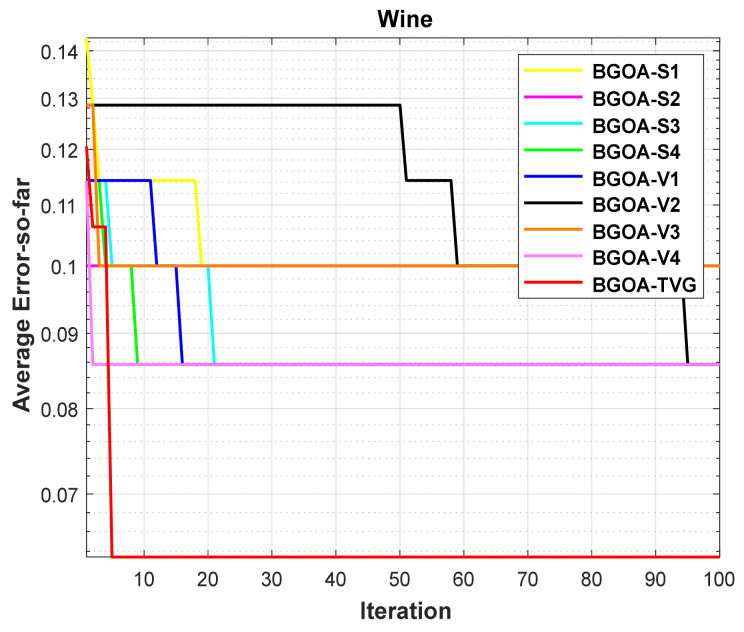
Wine.

**Figure 19:**
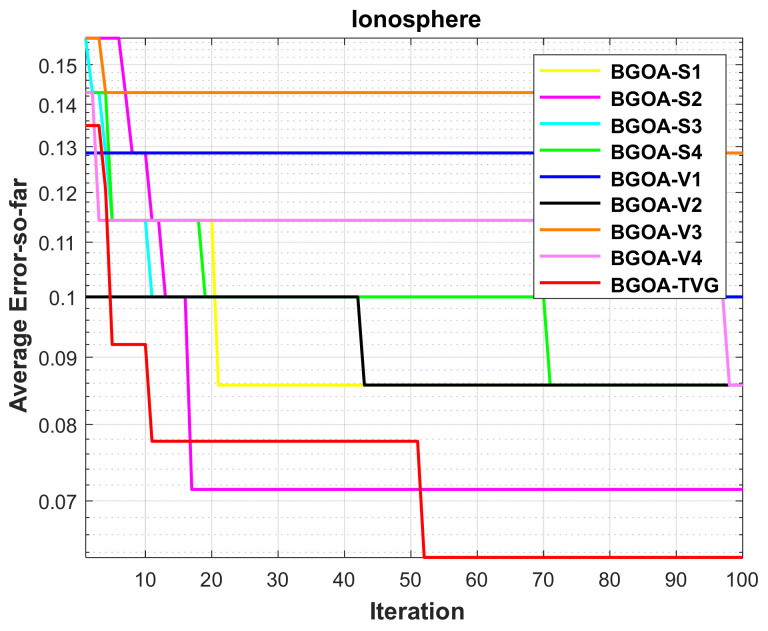
Ionosphere.

**Figure 20:**
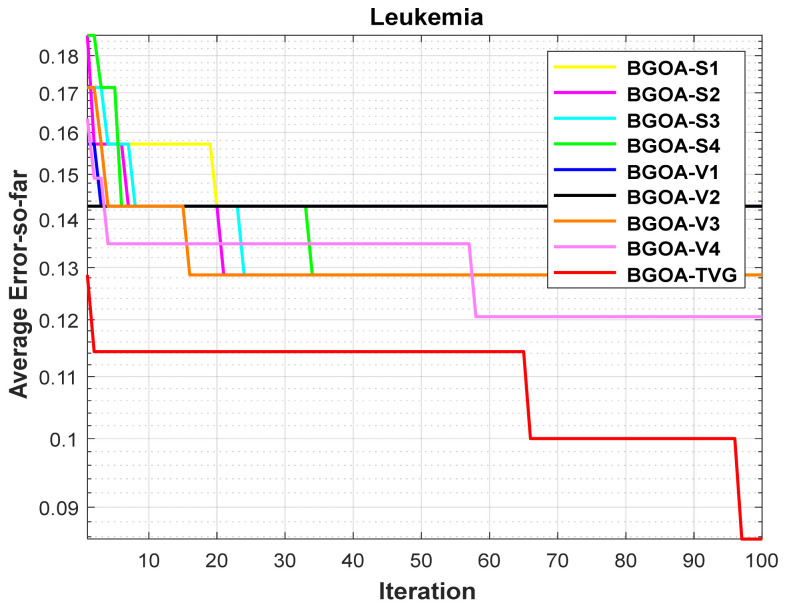
Leukemia.

**Figure 21:**
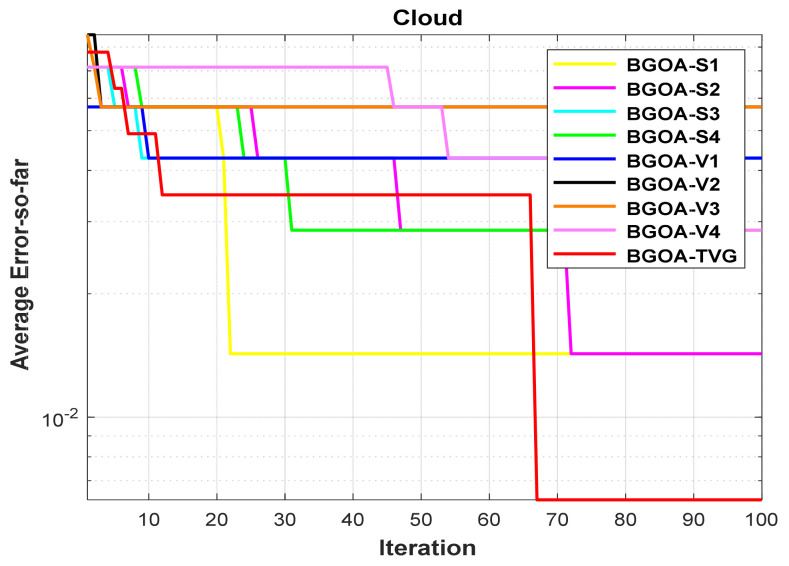
Cloud.

**Figure 22:**
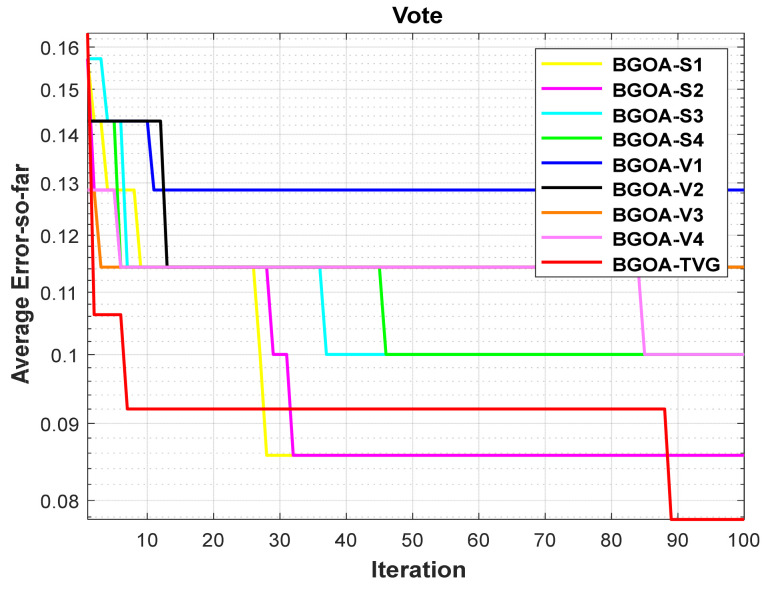
Vote.

**Figure 23:**
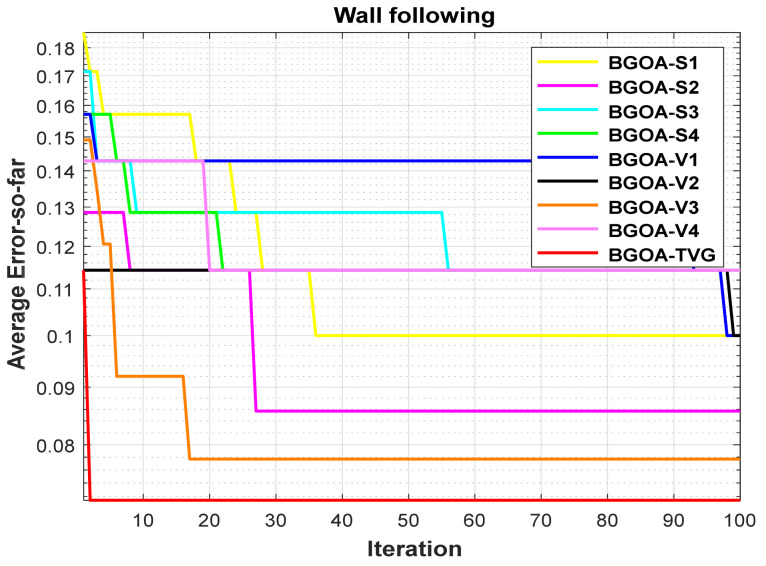
Wall-following robot navigation.

**Figure 24:**
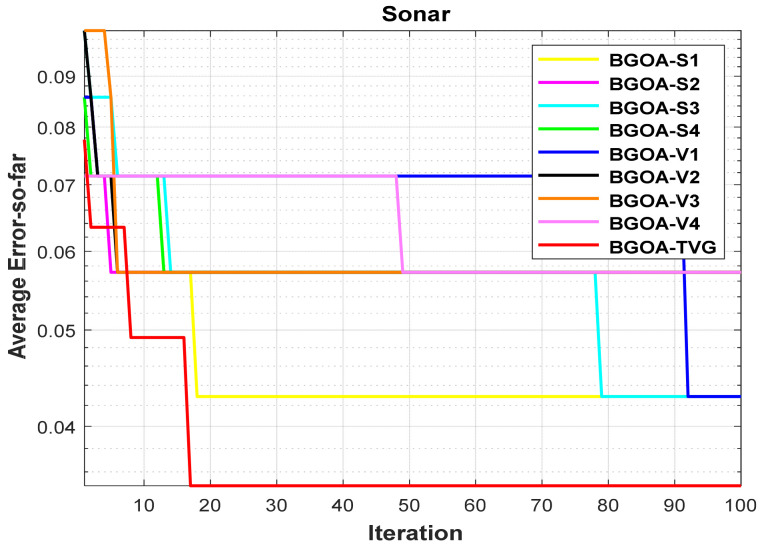
Sonar.

**Figure 25:**
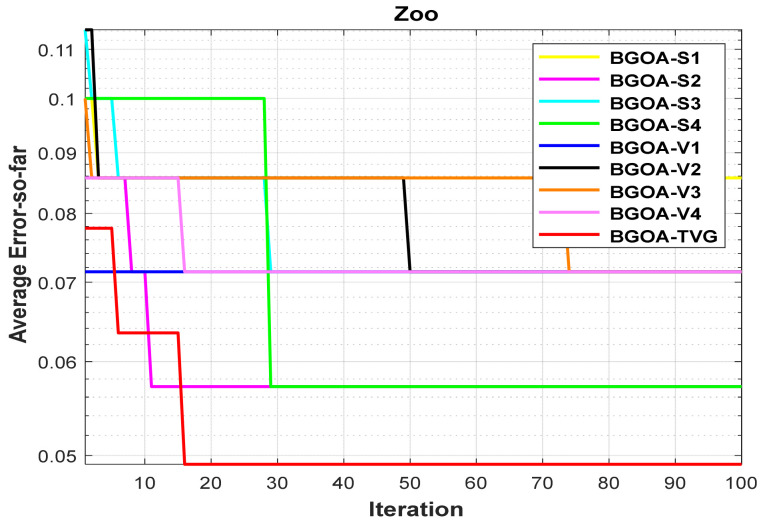
Zoo.

In order to demonstrate the impact of different types of conversion functions on the final data results, we selected four classic S-shaped transfer functions and four classic V-shaped transfer functions, which are shown in Table 4. We compared these with our proposed time-varying transfer function. The grasshopper optimization algorithm was used, and the results are shown in Figure 14, Figure 15, Figure 16, Figure 17, Figure 18, Figure 19, Figure 20, Figure 21, Figure 22, Figure 23, Figure 24 and Figure 25.

## 6. Electroencephalogram (EEG) Dataset Analysis

In this section, we present the proposed approach for channel selection for EEG-based signal acquisition. An EEG is very valuable for the diagnosis, identification, and treatment monitoring of epilepsy. Epilepsy often manifests itself as uncontrollable convulsions and involuntary behaviors. By observing and analyzing the signals recorded via EEGs, not only does it not harm a patient, but it can also help physicians determine if a patient has epilepsy through mapping analyses. In cases such as brain injury or stroke, EEGs can provide doctors with critical information to help them plan patients’ treatment. This is why EEGs have important clinical applications.

### 6.1. EEG Dataset

#### 6.1.1. DEAP Dataset

The EEG signals used in this work were obtained from the EEG Motor Movement/Imagery dataset. The data were collected from 109 healthy volunteers using the BCI2000 System, which makes use of 64 channels (sensors) and provides a separated EDF (European data format) file for each of them. The subjects performed different motor/imagery tasks. These tasks are mainly used in BCI (brain–computer interface) applications and neurological rehabilitation and consist of imagining or simulating a given action, like opening and closing one’s eyes, for example. Each subject performed four tasks according to the position of a target that appeared on the screen placed in front of them (if the target appears on the right or left side, the subject opens and closes the corresponding fist; if the target appears on the top or bottom, the subject opens and closes both fists or both feet, respectively). In short, the four experimental tasks were as follows:

1. To open and close their left or right fist;
2. To imagine opening and closing their left or right fist;
3. To open and close both their fists or both their feet;
4. To imagine opening and closing both their fists or both their feet.

Each of these tasks were performed three times, thus generating 12 recordings for each subject for a two-minute run, and the 64 channels were sampled at 160 samples per second. The features of the twelve recordings are extracted by means of an AR model with three output configurations for each EEG channel: 5, 10, and 20 features. Further, the average of each configuration is then computed in order to obtain just one feature per EEG channel (sensor). In short, for each sensor, we extracted three different numbers of AR-based features, with the output of each sensor being the average of their values. Henceforth, we have adopted the following notation for each of the dataset configurations: AR5 for the five autoregression coefficients extracted, and AR10 and AR20 for the ten and twenty autoregression coefficients, respectively. All the datasets we used were processed and can be found at https://openneuro.org/ (accessed on 31 January 2024).

#### 6.1.2. Dataset of EEG Recordings of Pediatric Patients with Epilepsy

Self-limiting epilepsy with central temporal spikes is a common focal epilepsy in childhood, mainly characterized by paroxysmal seizures in the mouth, pharynx, and on one side of the face. It is often accompanied by tongue stiffness, as well as speech and swallowing difficulties. Today, electroencephalography and other methods are the primary diagnostic tools for self-limiting epilepsy with central temporal spikes, and the prognosis is generally favorable. An epileptic electroencephalogram (EEG) refers to a special type of brain wave phenomenon induced by sleep, which is close to sustained spike slow-wave emissions and occurs more frequently during the SELECTS seizure period. In order to further investigate the impact of epileptic electrical persistence during sleep on patients’ pediatric symptoms, we selected the publicly available EEG dataset of pediatric epilepsy syndromes in a feature selection and accuracy analysis. In total, 88 subjects recorded EEGs with their closed eyes during the resting state in this dataset. A total of 36 of the participants were diagnosed with Alzheimer’s disease (AD group), 23 were diagnosed with frontotemporal fementia (FTD group), and 29 were healthy subjects (CN group). Using the international Mini-Mental State Examination (MMSE), their cognitive and neuropsychological state was measured. Lower MMSE scores indicate more severe cognitive decline, with scores ranging from 0 to 30. Months were used to measure the duration of the disease, with an average of 25 and an interquartile range (IQR) of 24 to 28.5 months. No comorbidities related to dementia were reported in the AD groups. The AD group experienced an average MMSE of 17.75 (SD = 4.5), while the FTD group had an average of 22.17 (SD = 8.22), and the CN group had an average of 30. The AD group averaged 66.4 years, the FTD group averaged 63.6 years, and the CN group averaged 67.9 years, which was the SD for the whole group. The data we used were analyzed and are now available at https://openneuro.org/ (accessed on 31 January 2024).

### 6.2. Compared Methods

The BGOA-TVG is an improved version of the grasshopper optimization algorithm (GOA) for multitask problems of pattern recognition. Furthermore, it is characterized as a swarm intelligence (SI) algorithm. SI has been proven to be a technique that can solve NP-hard computational problems, such as feature selection. Although a considerable number of new swarm-inspired algorithms have emerged in recent years, particle swarm optimization (PSO) is still the most widely used SI algorithm for solving feature selection problems [52]. In addition, the individual expression in SI for feature selection is typically a bit string, whereby the dimensionality of an individual is equal to the total number of features in the dataset. Binary encoding is more commonly used for feature selection than real encoding. Therefore, for feature selection, we compared the BGOA-TVG with BPSO [11,12], the BGA [24], BHHO [20], the BWOA [17], BACO [21] BGWO [14], and a method with all the features obtained from the time, frequency, and time–frequency domains. Their characteristics are shown in Table 5. All of three methods adopt 40 individuals for 100 iterations.

### 6.3. Classification Indices

In the experiment, five classification indices are used for the validation of the compared methods, including the true positive rate (recall, TPR), the positive predictive value (precision, PPV), the true negative rate (specificity, TNR), the negative predictive value (NPV), and the classification accuracy (ACC). They can be respectively defined as follows:

(21) $$TPR=\frac{TP}{TP+FN}$$

(22) $$PPV=\frac{TP}{TP+FP}$$

(23) $$TNR=\frac{TN}{TN+FP}$$

(24) $$NPV=\frac{TN}{TN+FN}$$

(25) $$ACC=\frac{TP+TN}{TP+TN+FP+FN}$$

### 6.4. Analysis of Results

#### 6.4.1. Selection of Classifier

**Figure 26:**
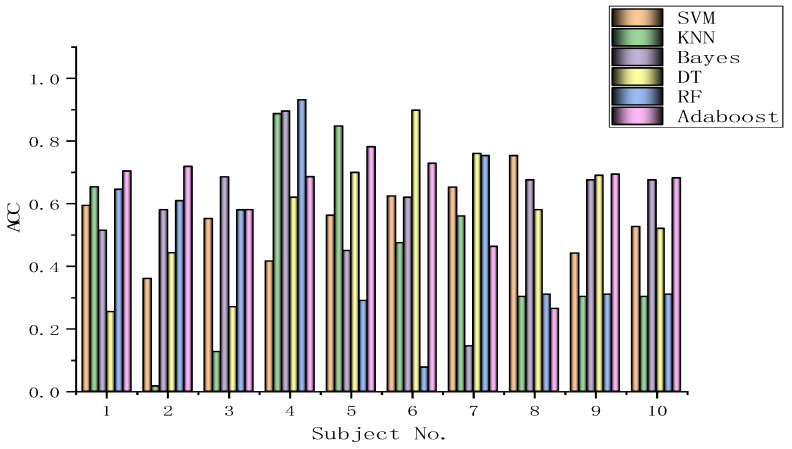
Classification accuracies of Allfeat.

**Figure 27:**
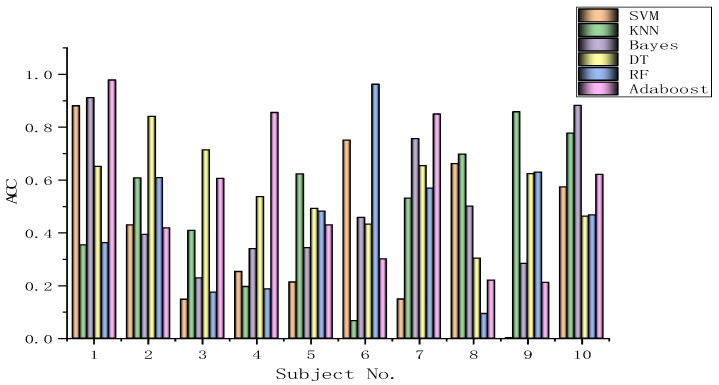
Classification accuracies of BPSO.

**Figure 28:**
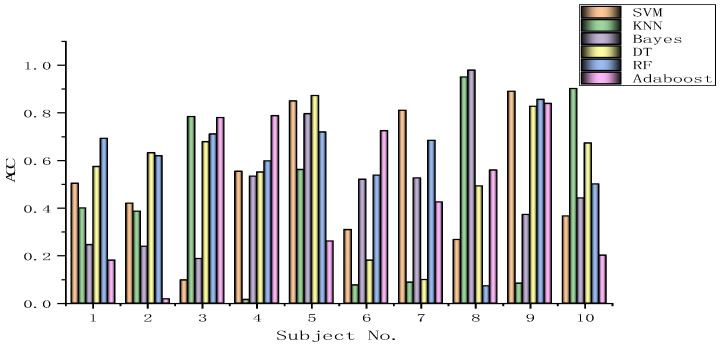
Classification accuracies of BGA.

**Figure 29:**
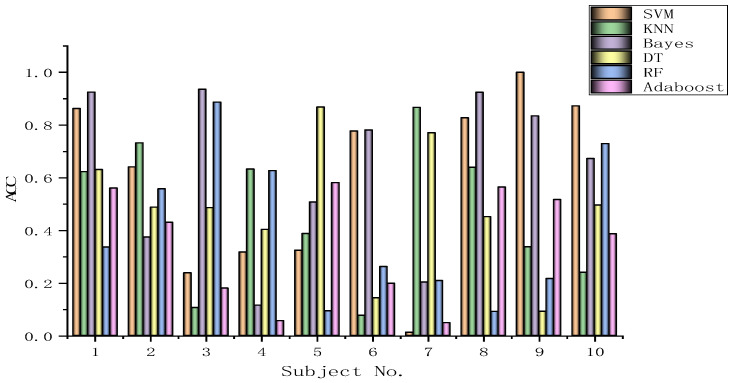
Classification accuracies of BHHO.

**Figure 30:**
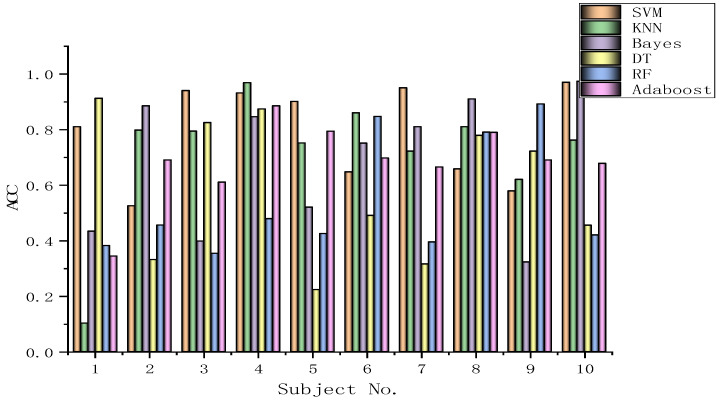
Classification accuracies of BWOA.

**Figure 31:**
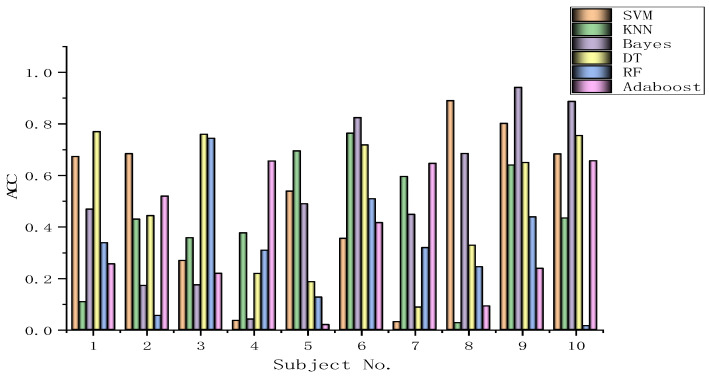
Classification accuracies of BACO.

**Figure 32:**
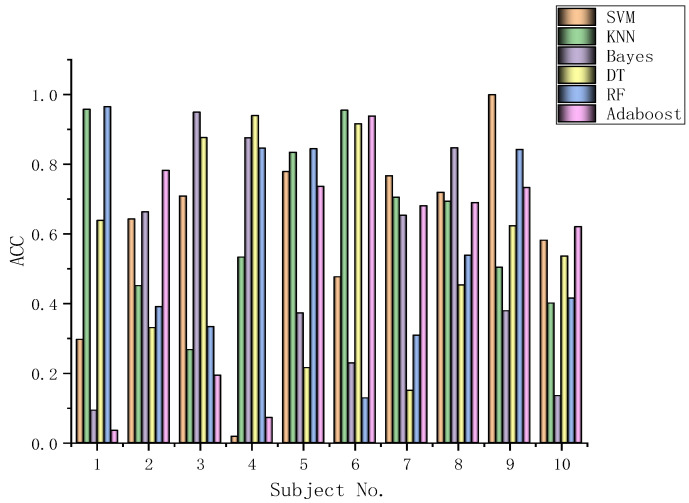
Classification accuracies of BGWO.

**Figure 33:**
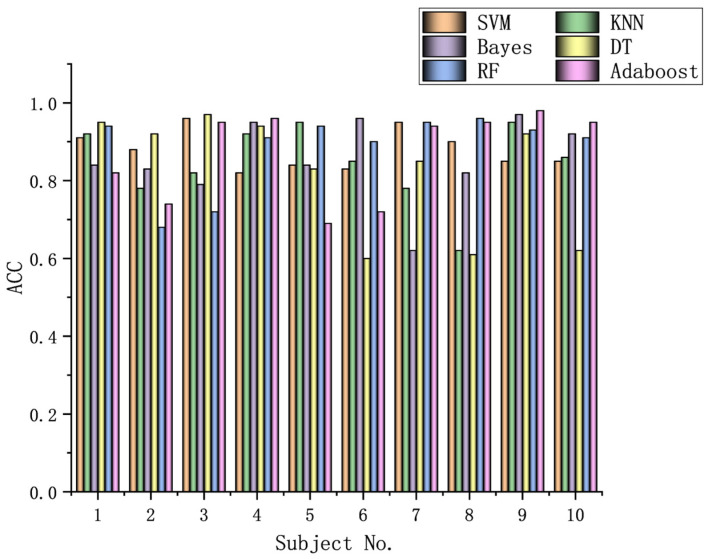
Classification accuracies of BGOA-TVG.

The algorithms chosen are directly applied to every subject dataset with a fixed classification method, i.e., the SVM, KNN, Bayes, DT, RF, or Adaboost methods. Their results for the DEAP dataset are recorded in Figure 26, Figure 27, Figure 28, Figure 29, Figure 30, Figure 31, Figure 32 and Figure 33, respectively. It can be seen that the classification accuracies of the five classifiers fluctuate prominently even for the same subject dataset and the same pattern recognition scheme. In terms of the highest classification accuracy, the best classifier is chosen artificially for every subject dataset, as shown in Table 6, respectively, corresponding to the DEAP dataset. It should be noted that the emotion recognition results for the same dataset depend on the classification method to some extent.

The training dataset comprises 50% of the original dataset, while 30% is allocated for the validation sets and the remaining 20% is reserved for the test sets. When considering the selection of classifiers for optimization, the BGOA-TVG undoubtedly realizes emotion recognition in a more efficient way. Moreover, according to the data in Figure 26, Figure 27, Figure 28, Figure 29, Figure 30, Figure 31, Figure 32 and Figure 33, the RF, DT, and Bayes methods are chosen as the best classifiers for emotion recognition with a high probability, while the KNN method is the worst one. This indicates that the RF, DT, and Bayes methods are more suitable for EEG-based emotion recognition than the KNN method.

#### 6.4.2. Selection of Features and Parameters

**Figure 34:**
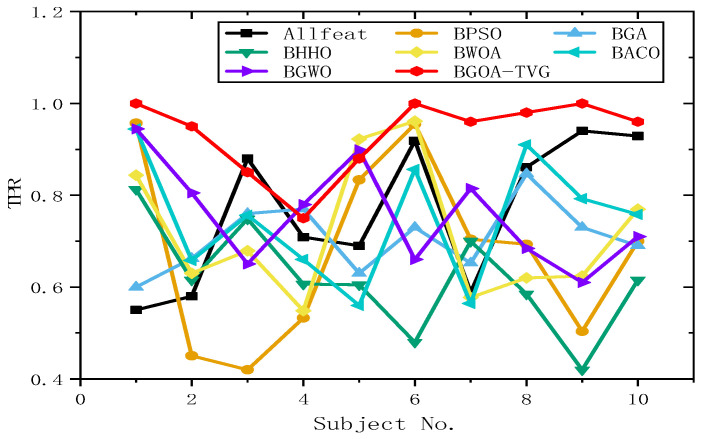
TPR obtained for ten subjects of DEAP dataset.

**Figure 35:**
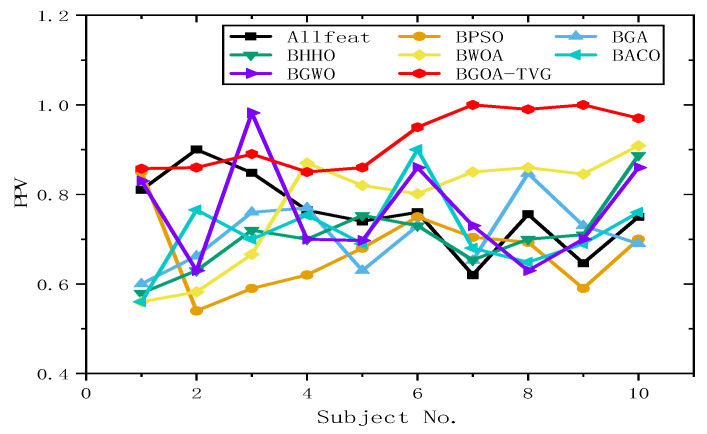
PPV obtained for ten subjects of DEAP dataset.

**Figure 36:**
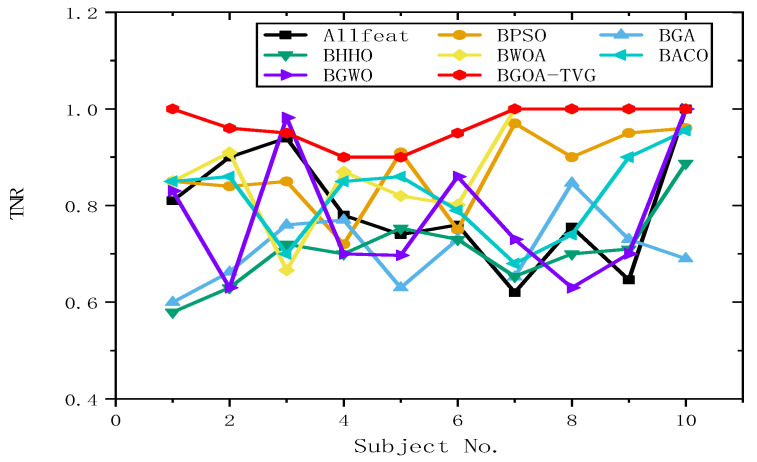
TNR obtained for ten subjects of DEAP dataset.

**Figure 37:**
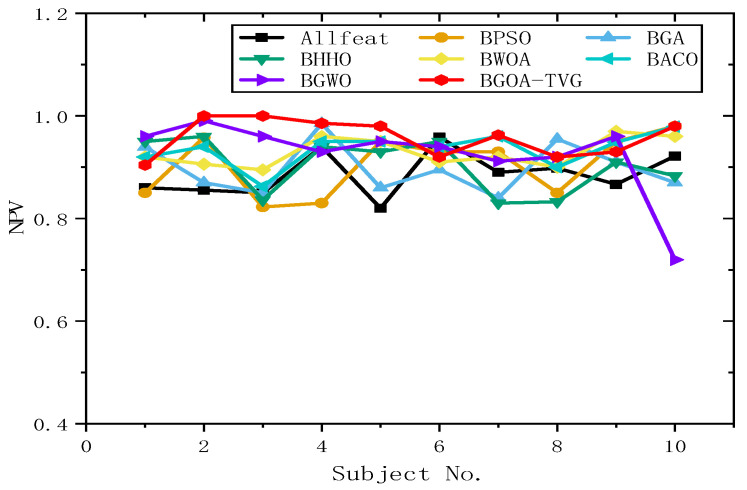
NPV obtained for ten subjects of DEAP dataset.

**Figure 38:**
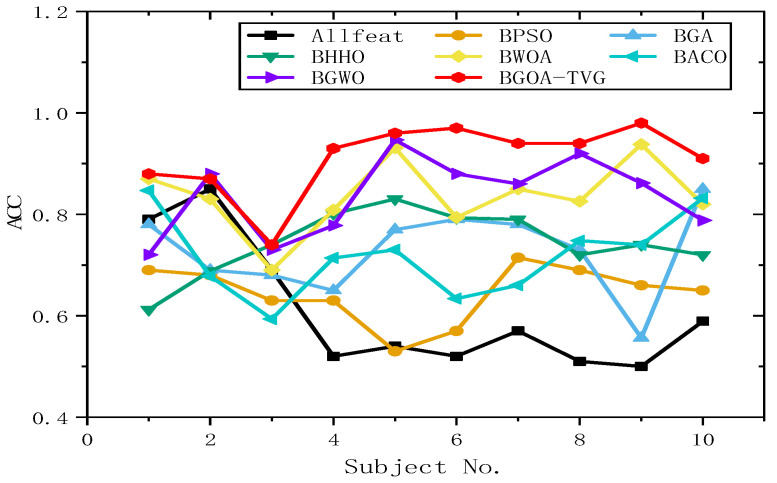
Mean ACC obtained for ten subjects of DEAP dataset.

To further identify the optimal results of the BGOA-TVG, we simultaneously recorded five classification indices for Allfeat, BPSO, the BGA, BHHO, the BWOA, BACO, and BGWO for all subject datasets. The parameters were the same as those in the previous experiment. The results are shown in Figure 34, Figure 35, Figure 36, Figure 37 and Figure 38. Firstly, the TPR (true positive rate), describes the ratio of all identified positive cases to all positive cases. It can be seen in Figure 34 that the classification index curve of the BGOA-TVG is better than the other algorithms most of time, and BPSO and BHHO have lower TPR values. Secondly, the PPV (positive predictive value) is shown in Figure 35. BGWO achieves the best performance in subject 3; however, it exhibits inferior results compared to BGOA-TVG in other subjects. Moreover, the volatility of the curve is too large, proving the poor robustness of the algorithm. In contrast, the BGOA-TVG is very robust. Thirdly, the TNR describes the proportion of identified negative cases to all negative cases. The BGOA-TVG shows the best robustness compared to the other algorithms, as can be seen in Figure 36. Fourthly, the NPV is the negative predictive value. In Figure 37, BGWO shows a similar classification rate as that of BHHO for subjects 1–9, but in subject 10, BGWO performs worse than the others, while BPSO, BACO, and the BGOA-TVG obtain a ratio of 1. Furthermore, the average classification accuracy values of the DEAP datasets found by these algorithms are recorded in Figure 38. It can been seen that the BGOA-TVG can choose an appropriate method based on the specific characteristics of an emotion dataset. The mean classification accuracy of the BGOA-TVG for DEAP is respectively higher than those of the other algorithms. This verifies that the BGOA-TVG can efficiently recognize emotion patterns.

#### 6.4.3. Analysis of Results

The horizontal lines in Figure 34, Figure 35, Figure 36, Figure 37 and Figure 38 represent the different datasets that we used, with subjects no. 1–10 corresponding to each of the ten datasets and the points representing the outcomes obtained using diverse algorithms. The lines indicate the compatibility and stability of the different algorithms. As a result, the proposed binary GOA is more stable than the other algorithms, and the application of the proposed algorithm for different DEAP datasets is universal.

**Figure 39:**
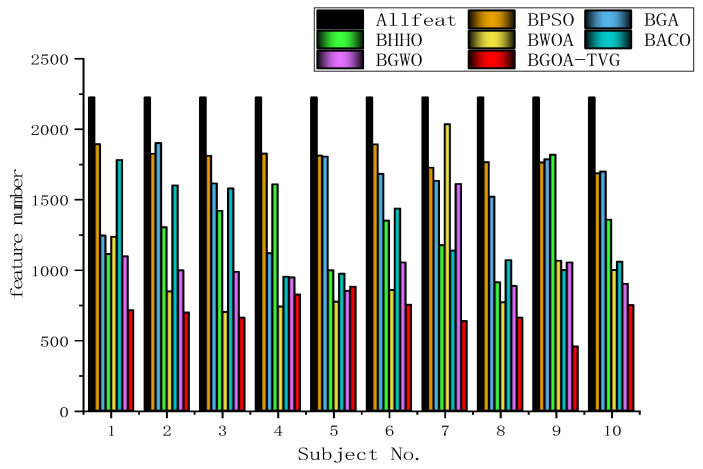
Mean feature number obtained for ten subjects from the DEAP dataset over forty iterations.

Figure 39 depicts the mean feature numbers for all optimization techniques regarding the learning algorithm. As we did not consider the feature extraction procedure, i.e., the autoregression coefficient computation, the feature numbers chosen over all dataset configurations are quite similar for BPSO and the BGA. In Table 7, it is possible to observe that the BGOA-TVG is the fastest technique in all situations, since it only updates one agent per iteration. Although this may be a drawback in terms of convergence, it is still the fastest approach with lowest number of features.

However, in Figure 38, the proposed binary GOA is the most accurate method compared to other algorithms and also selects fewer features. In general, the proposed algorithm can get results exhibit the most minimal error rate than other algorithms in EEG signal analysis.

Finally, we still need to deal with the trade-off between the number of features and the computational efficiency. Using all of the sensors does not lead to very different results, which supports the idea of this work: one can find a subset of sensors that can obtain reasonable results.

#### 6.4.4. Analysis of Results for Epilepsy EEG

**Figure 40:**
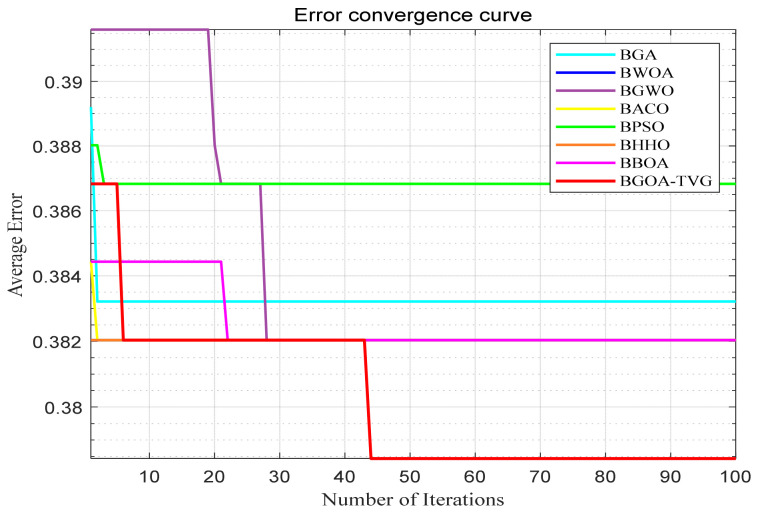
Sub-001.

**Figure 41:**
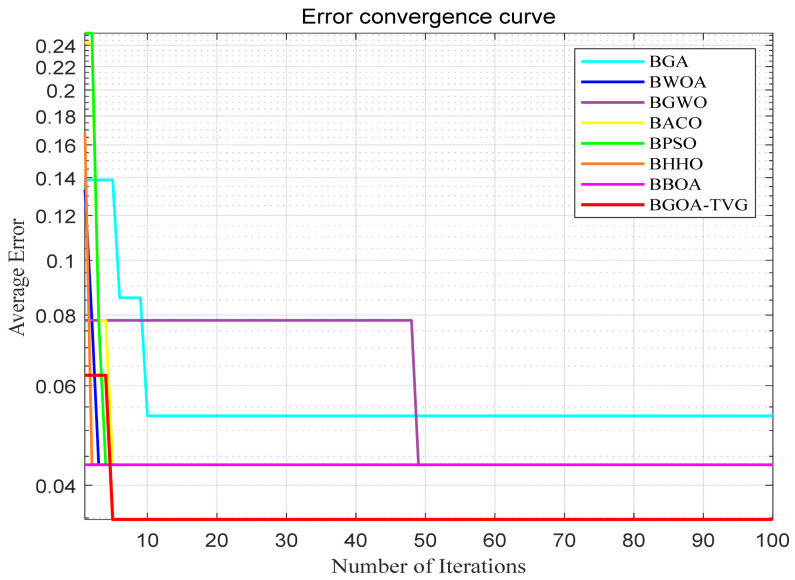
Sub-002.

**Figure 42:**
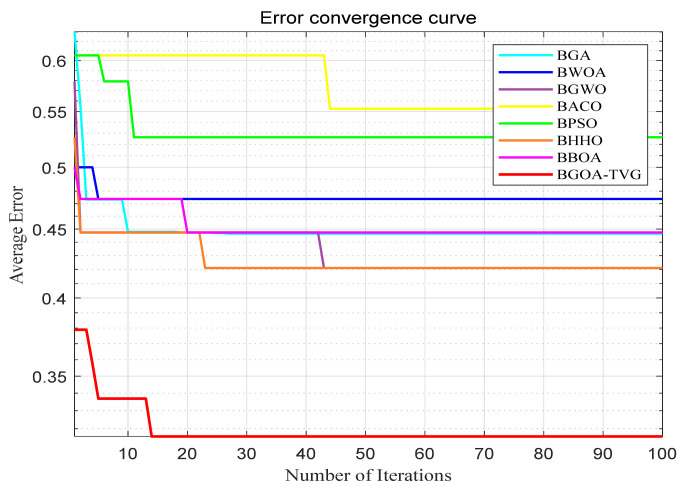
Sub-003.

**Figure 43:**
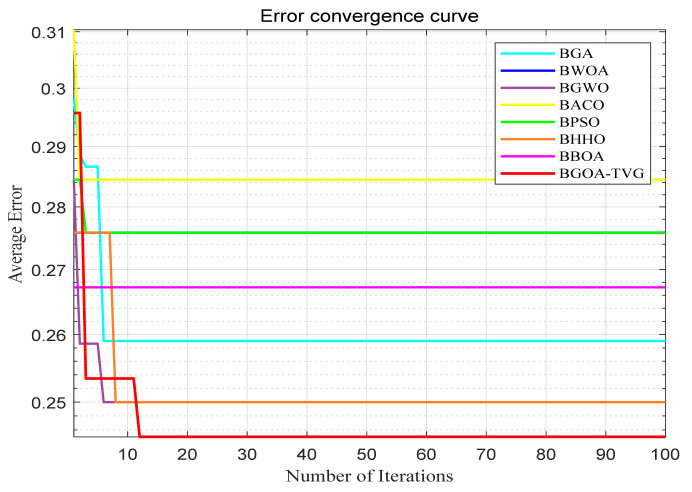
Sub-004.

**Figure 44:**
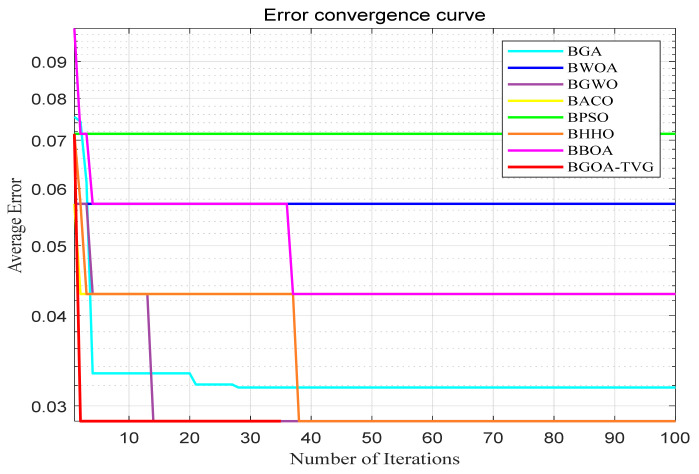
Sub-005.

**Figure 45:**
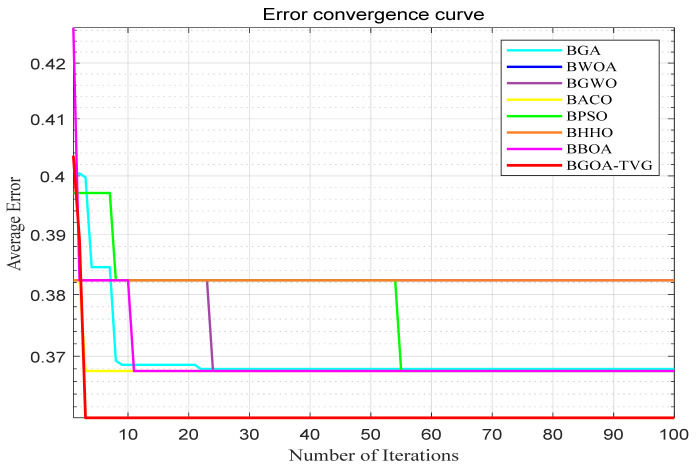
Sub-006.

**Figure 46:**
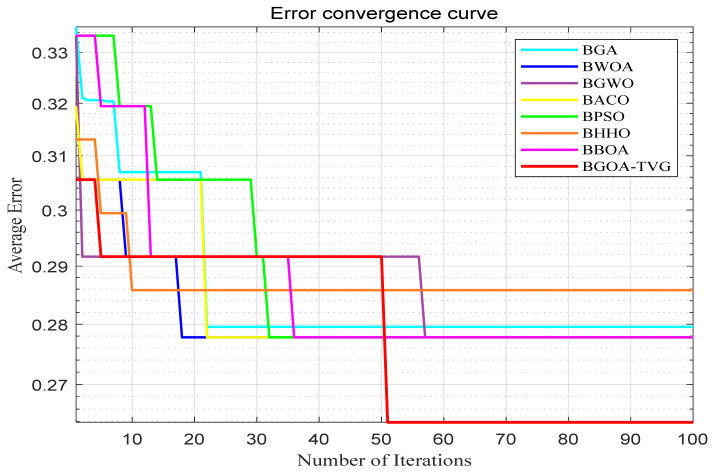
Sub-007.

**Figure 47:**
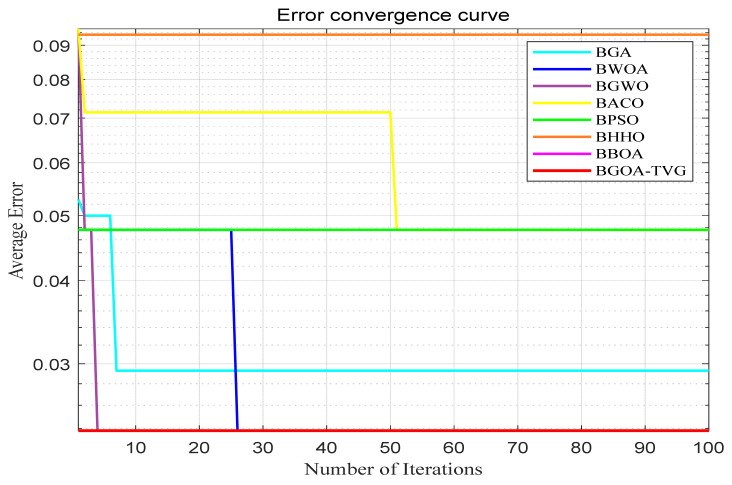
Sub-008.

**Figure 48:**
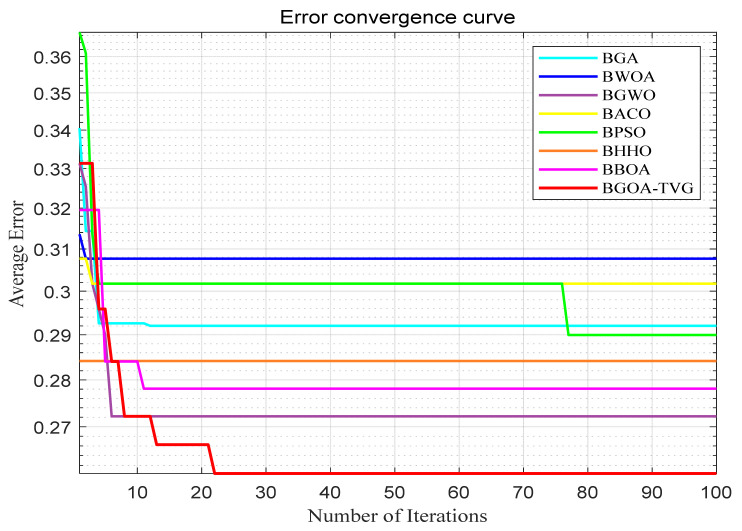
Sub-009.

**Figure 49:**
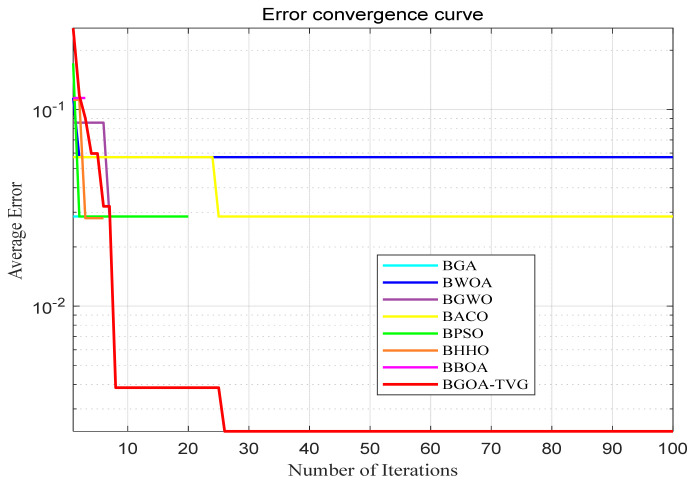
Sub-010.

**Figure 50:**
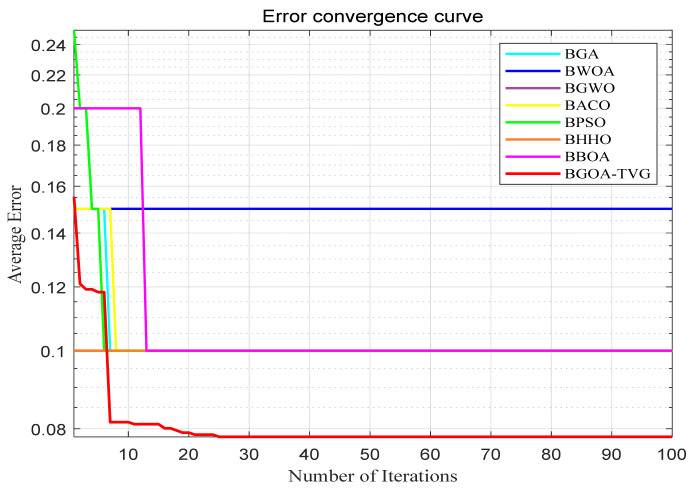
Sub-011.

**Figure 51:**
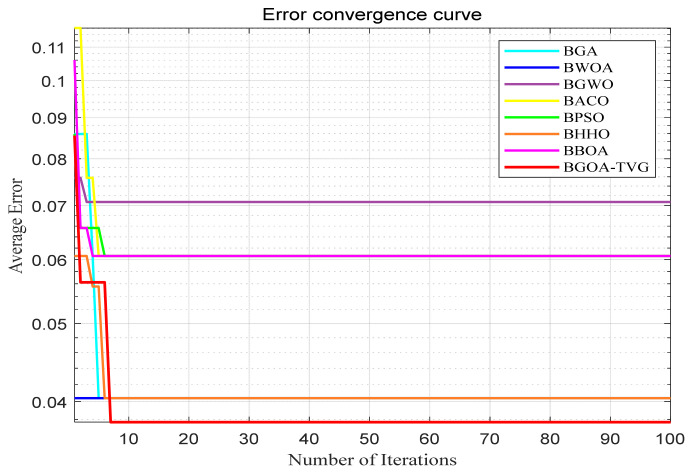
Sub-012.

Every subject dataset receives a direct application of the chosen algorithms through the SVM method, a fixed classification method. The epilepsy dataset results are documented in Figure 40, Figure 41, Figure 42, Figure 43, Figure 44, Figure 45, Figure 46, Figure 47, Figure 48, Figure 49, Figure 50 and Figure 51, respectively. The classification accuracy shows significant fluctuations for the various subject datasets. In terms of the highest classification accuracy, the best classifier is chosen artificially for every subject dataset. The results shown in Table 8 correspond to the epilepsy EEG datasets.

A total of 50% of the original dataset is present in the training dataset, while 30% and 20% are present in the validation and test sets, respectively. Without a doubt, the BGOA-TVG recognizes emotions in a more efficient manner. The data presented in Figure 39, Figure 40, Figure 41, Figure 42, Figure 43, Figure 44, Figure 45, Figure 46, Figure 47, Figure 48, Figure 49 and Figure 50 suggest that the BGOA-TVG is superior to the other algorithms when it comes to EEG-based emotion recognition.

## 7. Conclusions and Future Work

This paper proposed a new binary version of the grasshopper optimization algorithm, called the binary grasshopper optimization algorithm, using time-varying Gaussian mixed transfer functions (BGOA-TVG). Compared with the original GOA, which has a slow convergence speed and can easily fall into a local optimum, the BGOA-TVG has a good global search capability and accuracy in the local search space. Furthermore, the improved version of the GOA balances the relationship between exploration and exploitation in the search space and effectively avoids premature convergence. In order to verify the effectiveness and feasibility of the BGOA-TVG, 10 UCI datasets and a well-known DEAP dataset were tested for the algorithm. And the application of the K-nearest neighbor method further proves that the improved BGOA-TVG has a faster convergence speed in the global search space and a better accuracy in the local search space than other binary algorithms. For the BGOA-TVG, in future works, there are two possible research avenues. First, one could use different strategies to improve the original BGOA to increase the speed of searching the global space or obtain more accurate results in the local search space. Second, one could apply the BGOA-TVG to solve more complex optimization problems, such as image segmentation or network optimization configuration problems. Specific feature extraction applications are recommended for most mental illnesses, as they can quickly process a patient’s symptoms. Furthermore, augmenting the database volume will help us conduct more rapid and accurate data analyses.

## Figures and Tables

**Figure 4:**
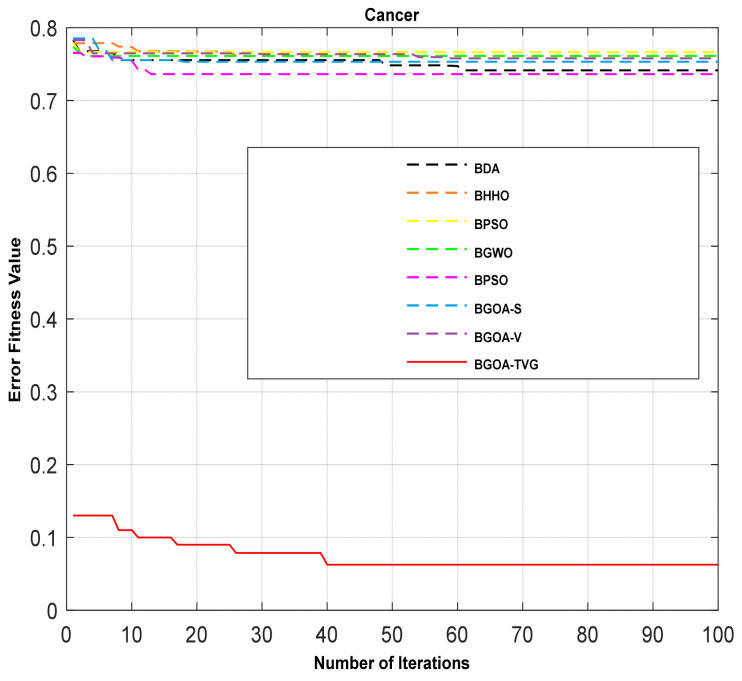
Cancer.

**Figure 5:**
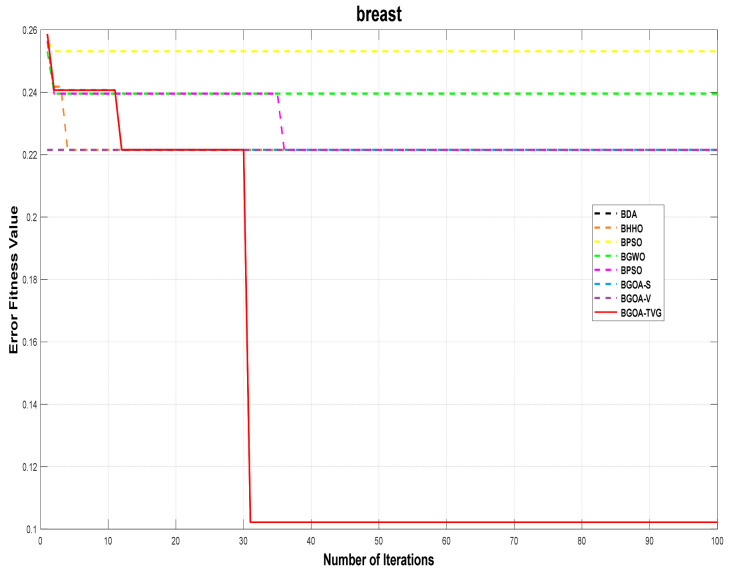
Breast.

**Figure 6:**
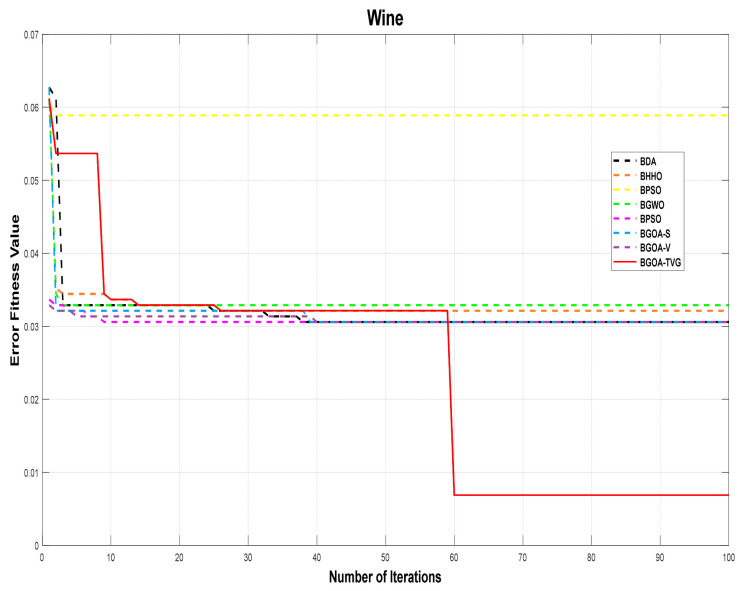
Wine.

**Figure 7:**
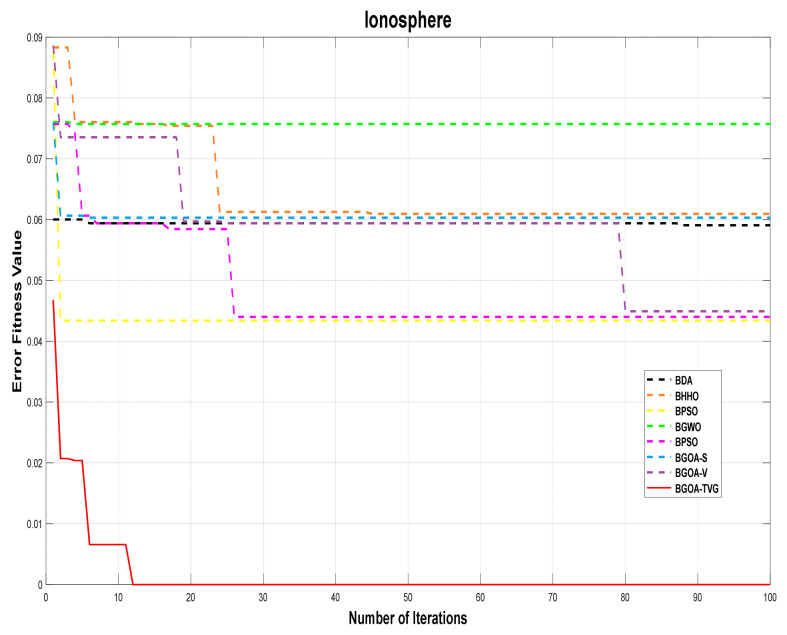
Ionosphere.

**Figure 8:**
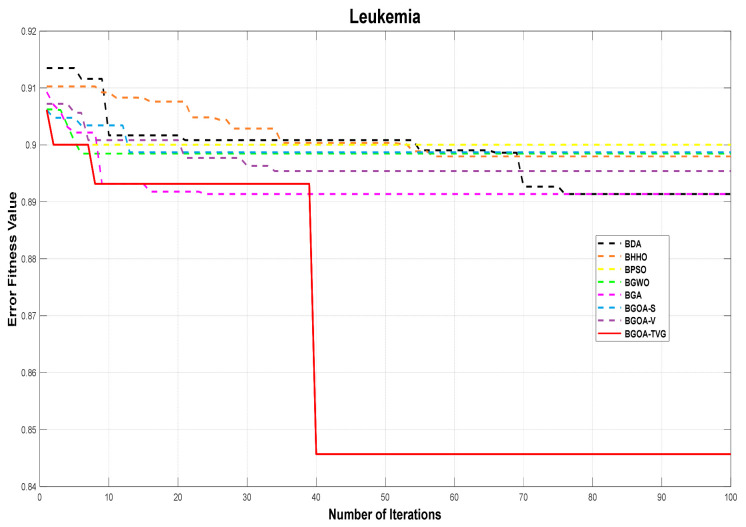
Leukemia.

**Figure 9:**
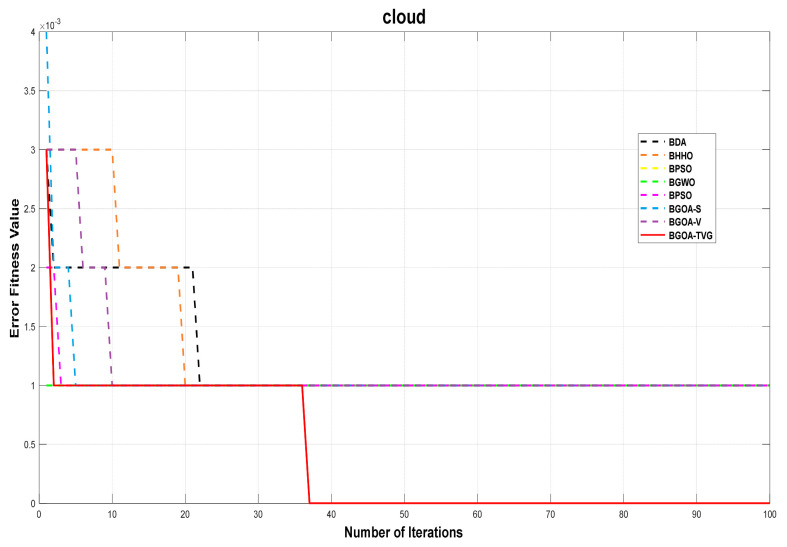
Cloud.

**Figure 10:**
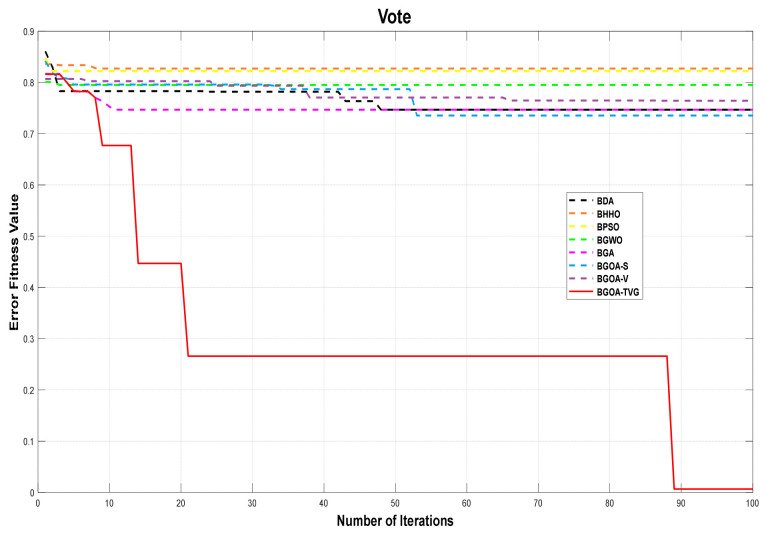
Vote.

**Figure 11:**
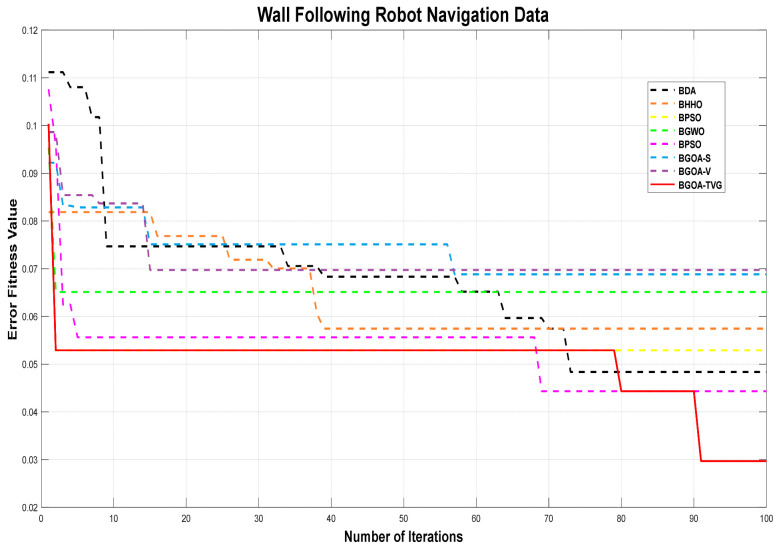
Wall-following robot navigation.

**Figure 12:**
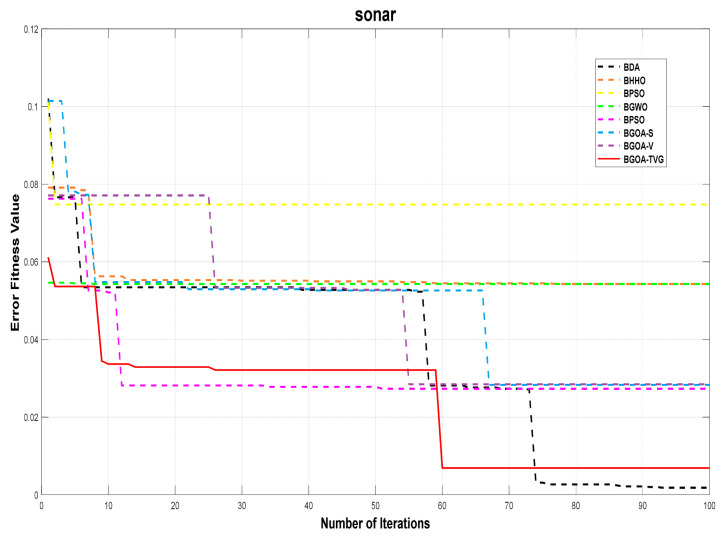
Sonar.

**Figure 13:**
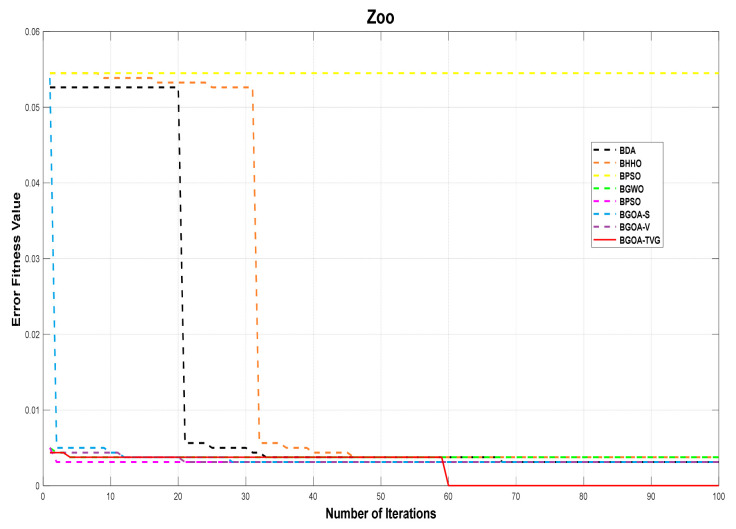
Zoo.

**Table 1:** UCI datasets.

No.	Dataset	Features	Instances
1	Cancer	9	683
2	Breast	9	277
3	Wine	13	178
4	Ionosphere	34	351
5	Leukemia	7129	72
6	cloud	10	1024
7	Vote	18	846
8	Wall following robot navigation data	24	5456
9	sonar	60	208
10	Zoo	16	101

**Table 2:** Parameter settings.

Algorithms	Parameter Values
BDA [23]	$W_{max}=0.9$; $W_{min}=0.4$
BHHO [20]	αu∈[2, 0]
BPSO [11]	$W_{max}=0.9$; $W_{min}=0.4$; $C_{1}=2$; $C_{2}=2$; $V_{max}=6$
BGWO [14]	αu∈[2, 0]
BWOA [17]	$\alpha u\in \left[2,\;0\right],\;\alpha =0.99,\;\beta =1$
BGBO [66]	Pr = 0.5
BGOA [65]	$iterval\in \left[0,\;2.079\right],\;l=1.5,\;f=0.5$
BGA [67]	*px* = 0.7, *G* = 0.95, *pm* = 0.01
BBOA [68]	*C* = 0.5, *α* = 0.1, *p* = 0.7
BGOA-TVG	$\alpha \in \left[0,\;5\right],\beta \in \left[0.05,\;10\right],\;sigma\in \left[0.01,\;10\right],\;iterval\in \left[0,\;2.079\right],\;l=1.5,\;f=0.5,\;C_{max}=1,\;C_{min}=0.00001$

**Table 3:** Test results.

Algorithm	Measure				
	Dataset	Average Fitness	Average Accuracy	Average Size	Rank
BDA [23]	Cancer	0.0106	0.9918	7.4000	2
BHHO [20]		0.0047	**1.0000**	14.0000	1
BPSO [11]		0.0324	0.9737	6.0000	6
BGWO [14]		0.0290	0.9743	10.6667	5
BGBO [66]		0.0017	**1.0000**	**4.1667**	1
BGA [67]		0.0131	**1.0000**	8.7667	1
BWOA [17]		0.0225	0.9820	14.1500	4
BGOA [65]		0.0025	0.9901	16.9000	3
BGOA-TVG		**0.0016**	**1.0000**	5.2000	1
BDA [23]	Breast	0.7580	0.2388	**2.5000**	4
BHHO [20]		0.6779	0.2381	8.0640	5
BPSO [11]		0.8230	0.1736	5.2000	6
BGWO [14]		0.6779	0.3211	7.0640	3
BGBO [66]		0.5047	0.4904	**2.5000**	2
BGA [67]		0.8710	0.1245	3.1619	7
BWOA [17]		0.8481	0.1498	7.5964	8
BGOA [65]		0.8366	0.1611	5.2265	9
BGOA-TVG		**0.8996**	**0.5698**	**2.5000**	1
BDA [23]	Wine	0.0056	0.9986	5.4500	2
BHHO [20]		0.0032	**1.0000**	4.1250	1
BPSO [11]		0.0687	0.9898	2.6667	3
BGWO [14]		0.0043	**1.0000**	5.6500	1
BGBO [66]		**0.0015**	**1.0000**	2.0667	1
BGA [67]		0.0034	**1.0000**	4.4000	1
BWOA [17]		0.0026	0.9764	5.1000	4
BGOA [65]		0.0039	**1.0000**	5.4000	1
BGOA-TVG		**0.0015**	**1.0000**	**1.9000**	1
BDA [23]	Ionosphere	0.0729	0.9291	9.0333	5
BHHO [20]		0.0719	0.9314	13.5000	4
BPSO [11]		0.0642	0.9391	12.9667	3
BGWO [14]		0.1172	0.8862	15.4333	8
BGBO [66]		**0.0326**	**0.9681**	**3.3667**	1
BGA [67]		0.0683	0.9314	7.8000	4
BWOA [17]		0.0890	0.9136	11.5500	6
BGOA [65]		0.0910	0.9119	12.9000	7
BGOA-TVG		0.4112	0.9441	4.2560	2
BDA [23]	Leukemia	0.0036	**1.0000**	2560.7667	1
BHHO [20]		0.1419	0.8595	1997.3000	2
BPSO [11]		0.1463	0.8571	3452.6333	3
BGWO [14]		0.0049	**1.0000**	3524.7667	1
BGBO [66]		**0.0000**	**1.0000**	**18.0333**	1
BGA [67]		0.0044	**1.0000**	3170.1000	1
BWOA [17]		0.0756	0.8119	3498.6333	4
BGOA [65]		0.1915	0.8119	3787.0667	4
BGOA-TVG		**0.0000**	**1.0000**	21.0565	1
BDA [23]	Cloud	0.4278	0.8076	467.9000	8
BHHO [20]		0.4937	0.8302	469.6667	5
BPSO [11]		0.6348	0.8377	466.1667	3
BGWO [14]		0.3956	0.8170	650.0333	7
BGBO [66]		0.1461	0.8393	53.4667	4
BGA [67]		0.4545	0.7843	429.6667	9
BWOA [17]		0.4973	0.9145	438.1000	2
BGOA [65]		0.4947	0.8226	502.3667	6
BGOA-TVG		**0.1065**	**0.9200**	**49.5760**	1
BDA [23]	Vote	0.0022	**1.0000**	3.5500	1
BHHO [20]		0.0176	0.9850	4.3500	5
BPSO [11]		0.0590	0.9667	**1.9000**	7
BGWO [14]		0.0034	0.9994	4.6333	2
BGBO [66]		**0.0013**	**1.0000**	2.0000	1
BGA [67]		0.0576	0.9456	5.9667	8
BWOA [17]		0.0109	0.9925	5.5500	3
BGOA [65]		0.0220	0.9822	7.1000	3
BGOA-TVG		0.0056	0.9906	2.2336	4
BDA [23]	Wall-Following Robot Navigation Data	0.0231	0.8076	8.2500	8
BHHO [20]		0.0183	0.8302	9.5000 s	5
BPSO [11]		0.1548	0.8377	5.1333	4
BGWO [14]		0.0485	0.8170	10.3667	7
BGBO [66]		0.0059	0.8393	5.0000	3
BGA [67]		0.1358	0.7843	7.9000	9
BWOA [17]		0.0206	0.9145	23.6842	2
BGOA [65]		0.0231	0.8226	12.1667	3
BGOA-TVG		**0.0055**	**0.9177**	**2.0000**	1
BDA [23]	Sonar	0.0582	0.9452	24.0667	5
BHHO [20]		0.0801	0.9235	26.0000	7
BPSO [11]		0.1547	0.9333	21.6000	6
BGWO [14]		0.0571	0.9460	22.0000	4
BGBO [66]		0.0543	0.9476	10.2222	3
BGA [67]		0.0523	0.9508	21.3667	2
BWOA [17]		0.1206	0.8833	30.7500	9
BGOA [65]		0.1182	0.8865	34.8333	8
BGOA-TVG		**0.0500**	**1.0000**	**9.5210**	1
BDA [23]	Zoo	0.0032	**1.0000**	5.1000	1
BHHO [20]		0.0034	**1.0000**	5.4000	1
BPSO [11]		0.1251	0.9400	4.4333	4
BGWO [14]		0.0029	**1.0000**	4.6667	1
BGBO [66]		0.0020	**1.0000**	**3.1053**	1
BGA [67]		0.0022	**1.0000**	3.5000	1
BWOA [17]		0.0035	0.9481	25.3000	3
BGOA [65]		0.0089	0.9967	9.0000	2
BGOA-TVG		**0.0009**	**1.0000**	4.0000	1

The best results are shown in bold.

**Table 4:** Transfer functions.

Name (S-Shaped Family)	Transfer Function
S1	$T\left(x\right)=\frac{1}{1+e^{-x}}$
S2	$T\left(x\right)=\frac{1}{1+e^{-2x}}$
S3	$T\left(x\right)=\frac{1}{1+e^{-\frac{x}{2}}}$
S4	$T\left(x\right)=\frac{1}{1+e^{-\frac{x}{3}}}$
Name (V-shaped family)	Transfer function
V1	$T\left(x\right)=\left|\frac{\sqrt{2}}{\pi }\int_{0}^{\frac{\sqrt{\pi }}{2}x}e^{-t^{2}}dt\right|$
V2	$T\left(x\right)=\left|\tanh(x)\right|$
V3	$T\left(x\right)=\left|\frac{x}{\sqrt{1+x^{2}}}\right|$
V4	$T\left(x\right)=\left|\frac{2}{\pi }\arctan(\frac{\pi }{2}x)\right|$

**Table 5:** Characteristics of compared methods.

Scheme	Optimization Algorithm	Individual Expression	Objective Function	Extracted Features	Classifier
Allfeat	No	-	TPR, PPV, TNR, NPV, ACC	Optimized	Optimized
BPSO	Yes	Binary	TPR, PPV, TNR, NPV, ACC	Optimized	Optimized
BGA	Yes	Binary	TPR, PPV, TNR, NPV, ACC	Optimized	Optimized
BHHO	Yes	Binary	TPR, PPV, TNR, NPV, ACC	Optimized	Optimized
BWOA	Yes	Binary	TPR, PPV, TNR, NPV, ACC	Optimized	Optimized
BACO	Yes	Binary	TPR, PPV, TNR, NPV, ACC	Optimized	Optimized
BGWO	Yes	Binary	TPR, PPV, TNR, NPV, ACC	Optimized	Optimized
BGOA-TVG	Yes	Binary	TPR, PPV, TNR, NPV, ACC	Optimized	Optimized

Optimized: means that the item value is not constant and chosen by the corresponding optimization algorithm.

**Table 6:** Best classifiers for ten subjects of DEAP obtained by different algorithms.

Subject No.	1	2	3	4	5	6	7	8	9	10
Allfeat [53]	RF	RF	RF	DT	RF	Bayes	Bayes	RF	RF	RF
BPSO [53]	DT	DT	RF	DT	DT	DT	DT	RF	DT	DT
BFA [53]	DT	DT	RF	DT	DT	Bayes	RF	RF	DT	DT
BGA	RF	DT	KNN	Adaboost	DT	Adaboost	SVM	Bayes	SVM	KNN
BHHO	Bayes	KNN	Bayes	KNN	DT	Bayes	KNN	Bayes	SVM	SVM
BWOA	DT	Bayes	SVM	KNN	SVM	KNN	SVM	Bayes	RF	Bayes
BACO	DT	SVM	DT	Adaboost	KNN	Bayes	Adaboost	SVM	Bayes	Bayes
BGWO	RF	Adaboost	Bayes	DT	RF	KNN	SVM	Bayes	SVM	Adaboost
BGOA-TVG	DT	DT	DT	Adaboost	KNN	Bayes	RF	RF	Adaboost	Adaboost

**Table 7:** Mean values of classification results of algorithms for DEAP data.

Algorithm	TPR	PPV	TNR	NPV	ACC	Feature Number
Allfeat [53]	0.6848	0.6209	0.7157	0.7786	0.7580	2224
BPSO [53]	0.8491	0.7814	0.7940	0.8647	0.8400	1087
BFA [53]	0.8760	0.9355	**0.8577**	0.8185	0.92	1088
BGA	0.7114	0.7862	0.7953	0.7116	0.6998	1065
BHHO	0.8569	0.7962	0.5685	0.623	0.86	1152
BWOA	0.8966	0.79	0.8410	0.869	0.93	752
BACO	0.7952	0.6559	0.6974	0.7665	0.745	1526
BGWO	0.8966	0.8663	0.8	0.8142	0.896	711
BGOA−TVG	**0.9116**	**0.9112**	0.8465	**0.852**	**0.942**	**698**

The best results are shown in bold.

**Table 8:** Test results.

Algorithm	Measure (Error Rate)				
	Dataset	Average Error	Minimum Error	Runtimes	Rank
BGA	Sub-001	0.2522	0.0006	30	**2**
BHHO [17]		0.1387	0.0064	30	**5**
BPSO [50]		0.0983	0.0114	30	**6**
BGWO		0.2435	0.0045	30	**4**
BBOA		0.0783	0.0121	30	**7**
BACO		0.1681	0.0007	30	**3**
BWOA [46]		0.1333	0.0165	30	**8**
BGOA−TVG		**0.0435**	**0**	30	**1**
BGA	Sub-002	0.5263	0.0039	30	**8**
BHHO [17]		0.4465	0.014	30	**6**
BPSO [50]		0.3947	0.0051	30	**5**
BGWO		0.5263	0.001	30	**3**
BBOA		0.4211	0.0185	30	**7**
BACO		0.4211	0.0026	30	**4**
BWOA [46]		0.4474	0.0007	30	**2**
BGOA−TVG		**0.2737**	**0.0006**	30	**1**
BGA	Sub-003	0.2845	0.0083	30	**7**
BHHO [17]		0.2982	0.0006	30	**4**
BPSO [50]		0.3017	0.0074	30	**6**
BGWO		0.3103	0.0094	30	**8**
BBOA		0.2845	0.0001	30	**3**
BACO		0.2759	0.007	30	**5**
BWOA [46]		**0.2672**	**0**	30	**1**
BGOA−TVG		0.2759	**0**	30	**1**
BGA	Sub-004	0.0714	0.0022	30	**4**
BHHO [17]		0.0333	0.0183	30	**7**
BPSO [50]		0.0429	0.0198	30	**8**
BGWO		0.0429	0.0002	30	**2**
BBOA		0.0286	0.0027	30	**5**
BACO		0.0429	0.0031	30	**6**
BWOA [46]		0.0571	0	30	**1**
BGOA−TVG		**0.0071**	0.0011	30	**3**
BGA	Sub-005	0.4118	0.0197	30	**8**
BHHO [17]		0.4004	0.0074	30	**5**
BPSO [50]		0.3821	0.0171	30	**7**
BGWO		0.3824	0.0157	30	**6**
BBOA		0.3971	0.0033	30	**3**
BACO		0.3824	0.0024	30	**2**
BWOA [46]		0.4265	0.0061	30	**4**
BGOA−TVG		**0.3821**	0.0010	30	**1**
BGA	Sub-006	0.3194	**0.0074**	30	**5**
BHHO [17]		0.3206	0.0114	30	**6**
BPSO [50]		**0.2917**	0.0261	30	**8**
BGWO		0.3056	0.0003	30	**2**
BBOA		0.3333	0.0038	30	**3**
BACO		0.3056	0.0043	30	**4**
BWOA [46]		0.3333	0.0177	30	**7**
BGOA−TVG		0.3056	**0**	30	**1**
BGA	Sub-007	0.0381	0.0053	30	**4**
BHHO [17]		0.0529	0.0258	30	**8**
BPSO [50]		0.2381	0.0113	30	**7**
BGWO		0.1952	0.0052	30	**6**
BBOA		0.0476	**0**	30	**1**
BACO		0.0338	0.0043	30	**5**
BWOA [46]		0.0238	0.0006	30	**3**
BGOA−TVG		**0.0176**	0.0005	30	**2**
BGA	Sub-008	0.8571	0.0119	30	**6**
BHHO [17]		0.2592	0.0026	30	**3**
BPSO [50]		0.0286	0.0083	30	**5**
BGWO		0.0571	0.0160	30	**8**
BBOA		0.1714	0.0122	30	**7**
BACO		0.0857	0.0028	30	**4**
BWOA [46]		0.1719	0.0013	30	**2**
BGOA−TVG		**0.0143**	**0.0006**	30	**1**
BGA	Sub-009	0.1000	0.0256	30	**8**
BHHO [17]		0.2018	0.0146	30	**7**
BPSO [50]		0.2000	0.0048	30	**3**
BGWO		0.1500	0.0142	30	**6**
BBOA		0.2500	0.0057	30	**4**
BACO		**0.1000**	**0.0018**	30	**1**
BWOA [46]		0.2000	0.0099	30	**5**
BGOA−TVG		0.1500	0.0019	30	**2**
BGA	Sub-010	0.2343	**0.0008**	30	**4**
BHHO [17]		0.1418	**0.0061**	30	**7**
BPSO [50]		0.1635	0.0132	30	**8**
BGWO		0.0661	0.0005	30	**3**
BBOA		0.0710	0.0047	30	**6**
BACO		0.1572	0.0008	30	**4**
BWOA [46]		0.0583	0.0004	30	**2**
BGOA−TVG		**0.0054**	**0**	30	**1**
BGA	Sub-011	0.0355	0.0011	30	**3**
BHHO [17]		0.0277	0.0043	30	**6**
BPSO [50]		0.0355	0.0114	30	**8**
BGWO		0.0796	0.0067	30	**7**
BBOA		0.1519	0.0014	30	**4**
BACO		0.1623	0.0027	30	**5**
BWOA [46]		**0.0066**	0.0004	30	**1**
BGOA−TVG		0.0084	0.0006	30	**2**
BGA	Sub-012	0.0679	0.0569	30	**4**
BHHO [17]		0.0760	0.0698	30	**5**
BPSO [50]		0.0120	0.0069	30	**3**
BGWO		**0.0025**	0.0005	30	**2**
BBOA		0.0844	0.0775	30	**6**
BACO		0.1903	0.0960	30	**7**
BWOA [46]		0.0088	0.1336	30	**8**
BGOA−TVG		0.0083	**0**	30	**1**

The best results are shown in bold.

## Data Availability

Data can be found at https://openneuro.org/ (accessed on 31 January 2024).
